# 17q12 deletion syndrome mouse model shows defects in craniofacial, brain and kidney development, and glucose homeostasis

**DOI:** 10.1242/dmm.049752

**Published:** 2022-12-13

**Authors:** Emily B. Warren, Juan A. Briano, Jacob Ellegood, Taylor DeYoung, Jason P. Lerch, Eric M. Morrow

**Affiliations:** ^1^ Department of Molecular Biology, Cell Biology and Biochemistry, Brown University, Providence, RI 02912, USA; ^2^ Department of Psychiatry and Human Behavior, Warren Alpert Medical School of Brown University, Providence, RI 02912, USA; ^3^ Center for Translational Neuroscience, Carney Institute for Brain Science and Brown Institute for Translational Science, Brown University, Providence, RI 02912, USA; ^4^ Mouse Imaging Centre (MICe), Hospital for Sick Children, Toronto, ON M5T 3H7, Canada; ^5^ Wellcome Centre for Integrative Neuroimaging, The University of Oxford, Oxford OX3 9DU, UK

**Keywords:** Copy number variants, 17q12, Neurodevelopmental disorders, Head development, Forebrain development, Renal cysts and diabetes syndrome

## Abstract

17q12 deletion (17q12Del) syndrome is a copy number variant (CNV) disorder associated with neurodevelopmental disorders and renal cysts and diabetes syndrome (RCAD). Using CRISPR/Cas9 genome editing, we generated a mouse model of 17q12Del syndrome on both inbred (C57BL/6N) and outbred (CD-1) genetic backgrounds. On C57BL/6N, the 17q12Del mice had severe head development defects, potentially mediated by haploinsufficiency of *Lhx1*, a gene within the interval that controls head development. Phenotypes included brain malformations, particularly disruption of the telencephalon and craniofacial defects. On the CD-1 background, the 17q12Del mice survived to adulthood and showed milder craniofacial and brain abnormalities. We report postnatal brain defects using automated magnetic resonance imaging-based morphometry. In addition, we demonstrate renal and blood glucose abnormalities relevant to RCAD. On both genetic backgrounds, we found sex-specific presentations, with male 17q12Del mice exhibiting higher penetrance and more severe phenotypes. Results from these experiments pinpoint specific developmental defects and pathways that guide clinical studies and a mechanistic understanding of the human 17q12Del syndrome. This mouse mutant represents the first and only experimental model to date for the 17q12 CNV disorder.

This article has an associated First Person interview with the first author of the paper.

## Teaser

**Summary:** We developed a mouse model of 17q12 deletion syndrome, which phenocopies many of the features of the human disorder, including renal dysfunction, craniofacial, glucose homeostasis and brain abnormalities.

## INTRODUCTION

Copy number variants (CNVs) are deletions or duplications of contiguous gene intervals that confer susceptibility to neurodevelopmental disorders (NDDs) through the dosage alteration of these genes (Autism Spectrum Disorders Working Group of The Psychiatric Genomics Consortium, 2017; Pescosolido et al., 2013; Sanders et al., 2015; Sebat et al., 2007; Takumi and Tamada, 2018). CNVs at the 17q12 locus, which include heterozygous deletions and duplications, are associated with incompletely penetrant and variably expressive neuropsychiatric and medical conditions (Mefford et al., 2016; Mitchel et al., 2016; Moreno-De-Luca et al., 2010; Rasmussen et al., 2016). Individuals with 17q12 deletion (17q12Del) syndrome present with NDDs, including autism spectrum disorders (ASD), schizophrenia and intellectual disability/developmental delay (Clissold et al., 2016; Moreno-De-Luca et al., 2010; Rasmussen et al., 2016; Roehlen et al., 2018), as well as medical phenotypes, the most prominent of which is renal cysts and diabetes syndrome (RCAD) (Clissold et al., 2015). RCAD has a spectrum of phenotypes that combines defects in renal development with hepatic abnormalities, including mature-onset diabetes of the young type five (MODY5) (Bellanné-Chantelot et al., 2004). In a recent large-scale study of CNV loci in neuropsychiatric disorders, the 17q12Del demonstrated the highest hazard ratio for ASD of all CNVs examined (Calle Sanchez et al., 2022).

Brain structural abnormalities have been reported in 17q12Del syndrome patients, including ventricular dilatation (Loirat et al., 2010), mild cerebral or cerebellar atrophy (Kasperaviciute et al., 2011; Nagamani et al., 2010), and atrophy, hyperintensities and sclerosis of the hippocampus (Kasperaviciute et al., 2011; Nagamani et al., 2010). Mild craniofacial dysmorphology has also been described in 17q12Del syndrome, including macrocephaly, up- or down-slanting palpebral fissures, and depression of the nasal bridge (Clissold et al., 2016; Palumbo et al., 2014; Rasmussen et al., 2016). Less frequently, these features include asymmetries of the eyes (Rasmussen et al., 2016) and/or face (Palumbo et al., 2014). Facial dysmorphologies have been observed in ASD (Callaghan et al., 2019; Miles et al., 2008; Miller et al., 2005; Shapira et al., 2019; Wong et al., 2014), schizophrenia (Deutsch et al., 2015; Hennessy et al., 2007; Moberg et al., 2004) and CNV disorders (Girirajan and Eichler, 2010; Grayton et al., 2012; Mak et al., 2021; McDonald-McGinn and Sullivan, 2011; Morris et al., 2015; Urraca et al., 2013). Oligogenic mechanisms underlying craniofacial anomalies have been characterized in the 16p11.2 CNV (Qiu et al., 2019), whereas brain structural and cytoarchitectural defects have been robustly delineated in the 22q11.2 CNV (Diamantopoulou et al., 2017; Ellegood et al., 2014). Moreover, the availability of mouse models in CNV disorders, such as 22q11.2, 16p11.2, 3q29 and others, have substantially advanced experimental studies examining disease mechanisms (Gokhale et al., 2019; Kumar et al., 2018; Pollak et al., 2022; Qiu et al., 2019; Rein and Yan, 2020; Rutkowski et al., 2021; Zinkstok et al., 2019).

To date, the mechanistic understanding of the 17q12 CNV disorders has been limited as there is a complete absence of experimental models. The 17q12 CNV interval includes 15 protein-coding genes, two of which have strong links to the known medical and neuropsychiatric conditions of 17q12Del syndrome, namely the transcription factors hepatocyte nuclear factor-1-beta (*HNF1B*) and LIM homeobox 1 (*LHX1*), respectively. Haploinsufficiency of *HNF1B*, which encodes a transcription factor critical for renal tubulogenesis (Ma et al., 2007), is likely to be causative of RCAD in 17q12 deletion patients, as loss-of-function mutations in *HNF1B* alone are autosomal dominant for renal abnormalities (Bellanné-Chantelot et al., 2004; Bingham et al., 2001; Edghill et al., 2006). *LHX1* also plays a critical role in embryogenesis, during which it is required for head formation (Fossat et al., 2015; McMahon et al., 2019; Shawlot and Behringer, 1995). In the nervous system, *LHX1* has defined roles in interneuron migration (Symmank et al., 2019), circadian neurobiology (Bedont et al., 2014; Hatori et al., 2014) and Purkinje cell differentiation (Lui et al., 2017; Zhao et al., 2007), and is expressed in subpopulations of Cajal–Retzius cells (Hochgerner et al., 2018; Miquelajauregui et al., 2010). Dissecting the interplay of haploinsufficiency of these putatively pathogenic genes with the other genes in the interval remains an essential challenge in the study of 17q12Del syndrome.

To disentangle the pathophysiological underpinnings of 17q12Del syndrome, we generated a mouse model of the human disorder by deleting the syntenic region at mouse locus 11qC. For simplicity in this study, we refer to the deletion mouse model as the 17q12Del mouse. We crossed the deletion onto two backgrounds, C57BL/6N (B6) and CD-1. We observed lethal abnormalities in head, craniofacial and forebrain development on the B6 background, precluding study of adult animals. Similar, but less penetrant and milder, craniofacial and brain abnormalities were observed on the CD-1 background, enabling the examination of adult animals, in which we additionally observed abnormalities in kidney development and glucose homeostasis. From this study, we demonstrate that this novel 17q12Del mouse mutant provides an important new tool to dissect mechanisms in this human CNV disorder, including areas of broad significance, such as variable expressivity and sex-specific effects in NDDs.

## RESULTS

### Generation of 1.2 Mb deletion on chromosome 11qC in mouse B6 strain using CRISPR/Cas9

**Fig. 1.**
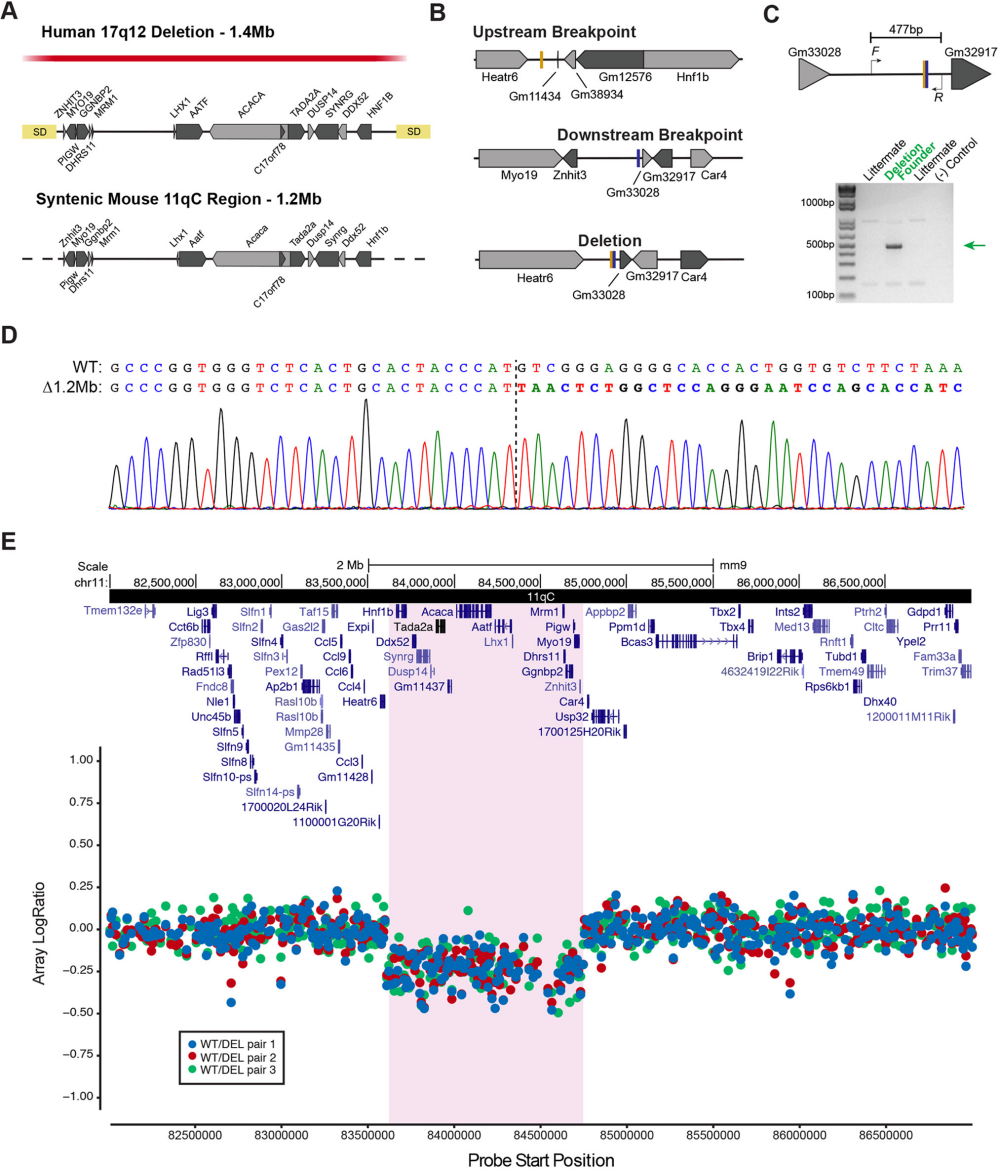
**Generation and validation of the 17q12 deletion (17q12Del) mouse.** (A) The human 17q12Del region and the aligned syntenic mouse 11qC genomic region. The red bar indicates the consensus human deletion region, spanning 1.4 Mb. The syntenic mouse locus is a 1.2 Mb region at 11qC. Note that the mouse locus is depicted in 3′-5′ orientation to show homology. (B­) sgRNAs were targeted to breakpoints upstream (5′ to protein-coding *Hnf1b* and non-coding *Gm11434*) (shown by gold hashmark on top) and downstream (3′ to *Znhit3*) (blue hashmark in middle) of the mouse 11qC locus. An ssDNA donor was introduced to facilitate the joining of the breakpoints. Bottom shows predicted CRISPR-mediated recombination yielding a 1.2 Mb deletion. (C) Forward and reverse primers were designed around the deletion junction to detect a 477 bp product in the presence of a deletion (top), confirmed by PCR (bottom, DNA agarose gel). Green arrow indicates deletion product. (D) Chromatogram of Sanger sequencing of PCR product. The chromatogram confirms the expected joined upstream and downstream sequences; dashed line indicates the breakpoint and deviation from the reference sequence. (E) Array comparative genomic hybridization of wild-type (WT) versus 17q12Del (DEL) littermates. Three pairs of WT/DEL offspring were used, and intensity log ratios reveal a consensus deletion covering the (highlighted) region between *Heatr6* and *Car4*. The aligned region is displayed with UCSC genes (reference assembly mm9).

The human 17q12Del interval spans a consensus 1.4 Mb interval, covering 15 protein-coding genes and flanked by segmental duplication regions (Fig. 1A). The protein-coding genes comprise *AATF*, *ACACA*, *C17orf78*, *DDX52*, *DHRS11*, *DUSP14*, *GGNBP2*, *LHX1*, *HNF1B*, *MRM1*, *MYO19*, *PIGW*, *SYNRG* and *ZNHIT3*. We sought to develop a mouse model with strong genetic construct validity to the human 17q12Del CNV disorder. The mouse 11qC locus has a 1.2 Mb region syntenic to the human 17q12 locus, including the same 15 protein-coding genes (Fig. 1A), although the interval is oriented in the reverse 5′-3′ direction relative to the human interval. Hereafter, we refer to the 11qC deletion mouse model as the 17q12Del mouse. We used a CRISPR/Cas9-mediated strategy to generate the 17q12Del mouse on the B6 background. We designed single-guide RNAs (sgRNAs) to points upstream of *Hnf1b* and downstream of *Znhit3* to cover the full interval. These were introduced along with a single-stranded DNA (ssDNA) donor that covered both regions to facilitate linking of the joined region. Successful deletion of the region removed all genes downstream of *Gm11434* and upstream of *Gm33028* (Fig. 1B). To detect the generation of the deletion, we designed primers around the breakpoint (Fig. 1C, top), which would amplify a 477 bp product only if the 1.2 Mb region was deleted (Fig. 1C, bottom). Once we generated a founder carrying the deletion, we validated the sequence of the PCR product using Sanger sequencing (Fig. 1D). Using these genome-editing methods, we produced a male founder with germline transmission of the 17q12Del mutation. After propagating this mutation, we validated the complete and consistent deletion of the full interval using array comparative genomic hybridization (aCGH). We used three pairs of B6:CD-1 F1 wild-type (WT) and 17q12Del littermates (see below) on a full chromosomal array and confirmed a consensus deletion interval at the 11qC locus in all three littermate pairs (Fig. 1E) that spanned the targeted region from *Hnf1b* to *Znhit3*.

### Survival to weaning of 17q12Del mice is dependent on the mouse background strain

**Fig. 2.**
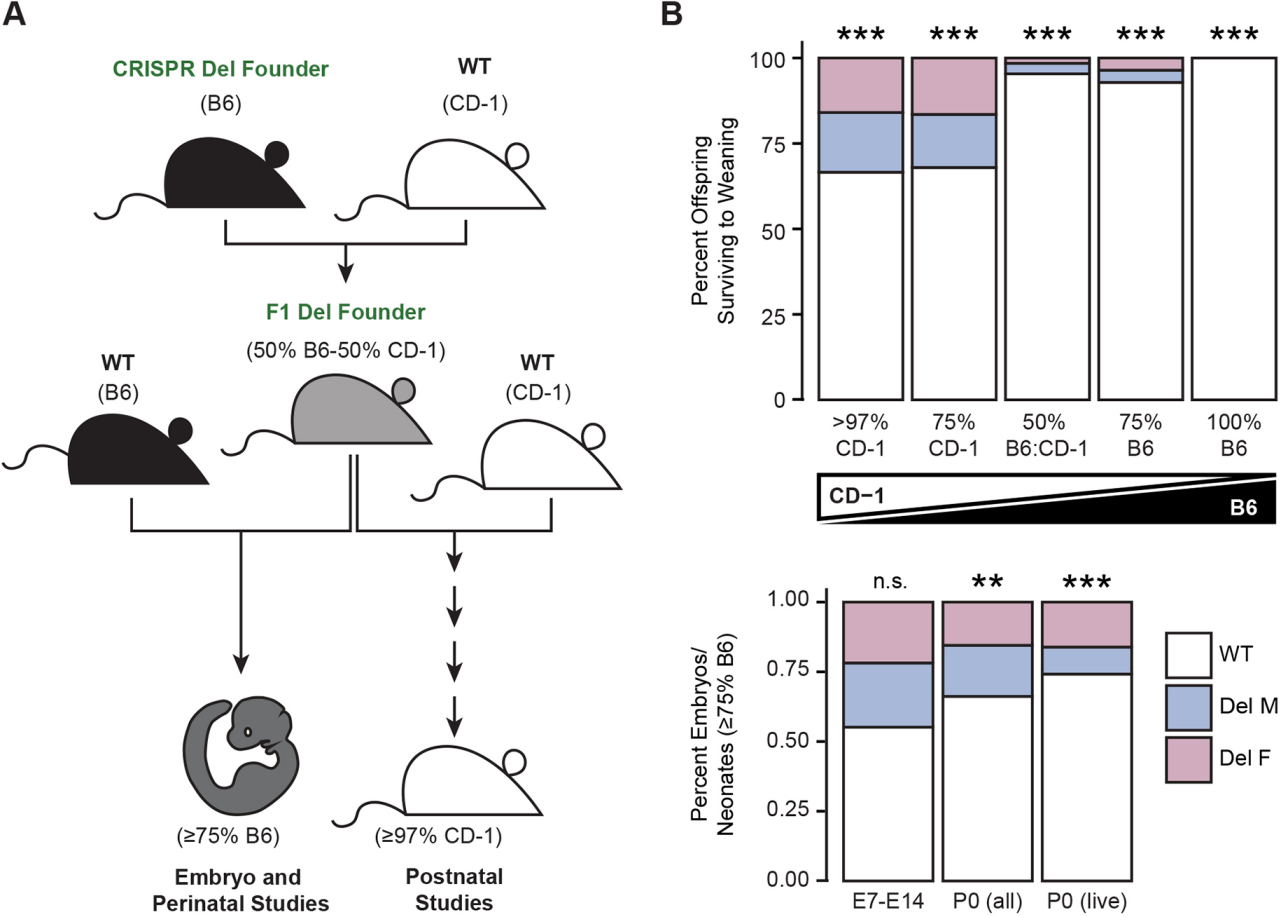
**Variable expressivity of the 17q12Del on two genetic backgrounds.** (A) Breeding schematic for propagating the 17q12Del mouse. Generation of the 17q12Del mouse was initiated on the C57BL/6N (B6) background (see Fig. 1). Attempts to propagate the mutation on a pure B6 background were unsuccessful due to lethality. The F1 deletion founder was generated by outcrossing the B6 17q12Del founder onto the outbred CD-1 strain. This F1 mixed background founder was backcrossed onto B6 for embryonic developmental studies, and further outcrossed onto CD-1 (F5+ generations, ≥97% CD-1 background) for postnatal studies. (B) Genetic background strongly affects the viability of 17q12Del mice. Top: increasing proportions of 17q12Del offspring survive to weaning with increasing percentages of CD-1 genetic background. Bottom: prenatally (up to E14), 17q12Del embryos (with ≥75% B6 background) are present at Mendelian ratios, but the number of viable offspring drops immediately at birth. See Table S1 for animal numbers. Chi-squared odds ratio test for significance. **P<0.001, ***P<0.001; n.s., not significant.

Importantly, we found distinct phenotypes on different mouse genetic backgrounds. We generated the 17q12Del founder on a B6 background; however, no live 17q12Del pups were born when attempting to maintain the mutation on this inbred, isogenic line. We outcrossed the founder onto the outbred CD-1 background (Fig. 2A), which, in addition to increased genetic heterogeneity, has a large litter size (Tanaka, 1998). This allowed us to derive mixed background F1 17q12Del founders. We attempted to re-backcross the deletion mutation onto the B6 line but observed a very high rate of head malformations in newborn offspring and were unable to maintain more than a very small number of live 17q12Del offspring with ≥75% B6 background. To study adult 17q12Del animals, we continued to outcross the 17q12Del mutation onto the CD-1 background to F5+ generations, or >97% CD-1 background identity. For the studies reported here, we used 17q12Del animals with ≥75% B6 background to explore prenatal to perinatal developmental phenotypes of the 17q12Del mutation, and animals with >97% CD-1 background to examine adult (6-8 weeks) phenotypes. See Fig. 2A for schematic outline and the Materials and Methods for details on the breeding strategy. For clarity, in subsequent figures we will refer to ≥75% B6 animals as ‘B6’, and >97% CD-1 animals as ‘CD-1’, unless otherwise specified.

We quantified the Mendelian ratios of deletion offspring surviving at weaning [postnatal day (P)21] during the outcrossing (to CD-1) and backcrossing (to B6) of the 17q12Del mutation (Fig. 2B). As described above, we observed no viable offspring from the 17q12Del mutation on a 100% B6 background, but a small number of offspring survived on a 50% or 75% B6 background. As outcrossing reached a majority CD-1 background, we observed a greater proportion of 17q12Del offspring (∼33%) surviving to weaning. However, this proportion remained significantly sub-Mendelian (chi-squared odds ratio test; 75% CD-1, χ^2^=13.291, *P*=0.0003; >97% CD-1, χ^2^=43.333, *P*=4.617×10^−11^) (Fig. 2B, top). In all ≥50% B6 backgrounds, many of the surviving offspring subsequently died or were euthanized at ∼6-8 weeks from head malformation-related complications, but all animals surviving to P21 are included in the calculations. The incidence of >97% CD-1 17q12Del animals dying or requiring euthanasia after weaning was 2.3% (see Materials and Methods).

We next investigated the timing of the 17q12Del lethality on the B6 background in greater detail. As we observed a high rate of neonatal lethality in B6 17q12Del embryos, we separately quantified the percentage of all newborn P0 B6 17q12Del offspring, as well as the percentage of P0 B6 17q12Del offspring recovered alive (Fig. 2B, bottom). Although both conditions still reflect sub-Mendelian ratios of mutant offspring (all offspring, χ^2^=7.4507, *P*=0.0063; live offspring, χ^2^=14.516, *P*=0.0001), the smaller percentage of live pups recovered suggests that some of the 17q12Del lethality on B6 background occurs after birth, but in the early neonatal period. Importantly, we did not observe significant perinatal lethality on the CD-1 background. To determine whether B6 17q12Del mice have prenatal lethality, we examined B6 embryos from embryonic day (E)7 to E14. We observed a ratio of B6 17q12Del embryos to B6 WT not significantly different from the Mendelian ratio (χ^2^=1.752, *P*=0.1857) (Fig. 2B, bottom), suggesting that the majority of B6 17q12Del lethality occurs between the late prenatal (after E14) and early postnatal stages. Across all backgrounds and developmental stages, we did not observe a significant difference in the proportion of male or female 17q12Del offspring. See Table S1 for all animal numbers.

### Embryonic head and brain developmental defects in 17q12Del mice are more severe in males

#### Embryonic development

**Fig. 3.**
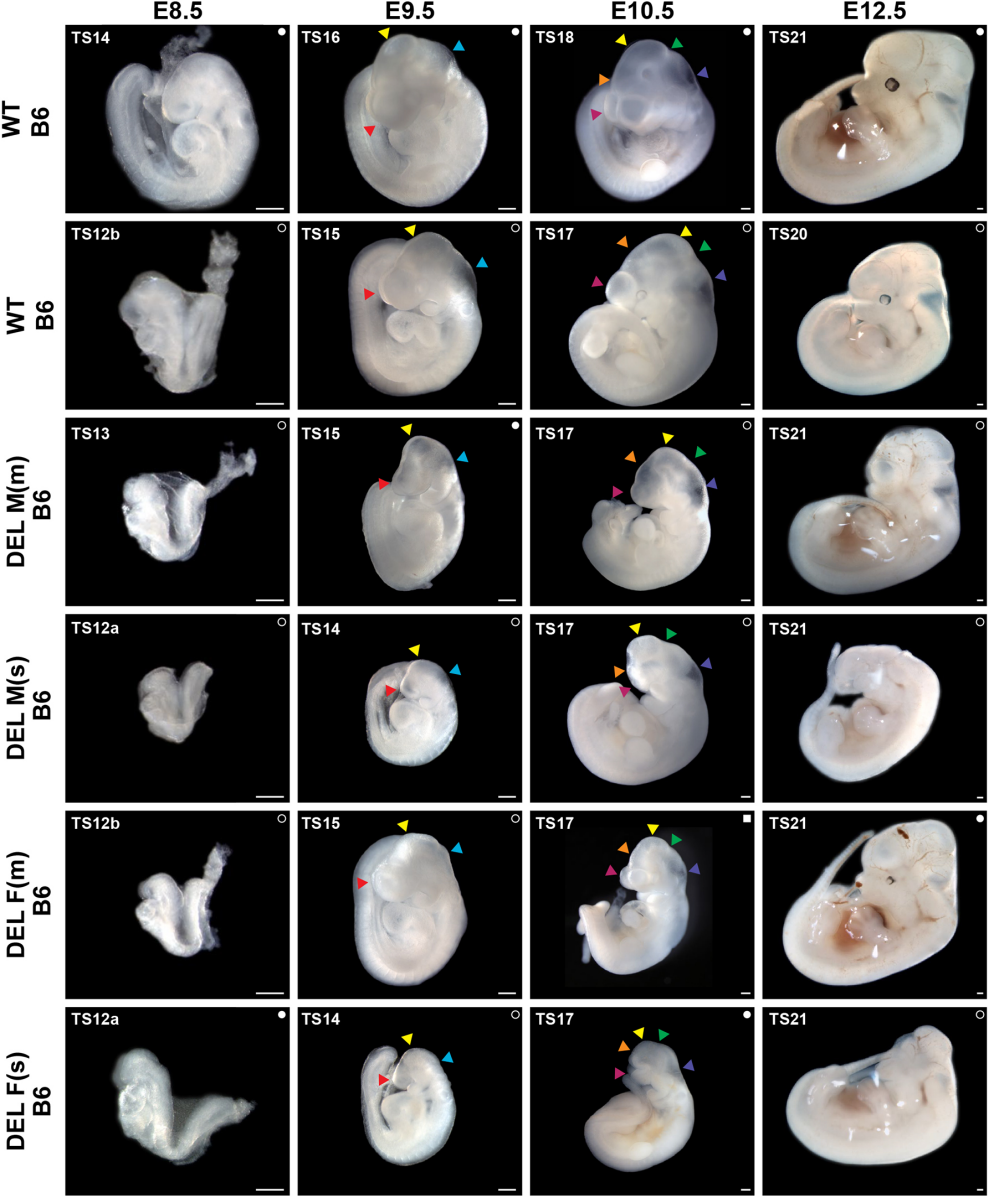
**Anterior malformations of B6 17q12Del embryos.** Representative male and female B6 17q12Del (DEL) embryos from E8.5 to E12.5 compared to WT littermates. ‘m’ embryos are representative examples of mild abnormalities; ‘s’ embryos are representative examples of severe abnormalities. Theiler stage (TS) for each embryo is indicated in the top-left corner, and litter for embryos at each time point is represented by a symbol in the top-right corner (i.e. filled circle, litter 1; open circle, litter 2). See Table S2 for details about embryo scoring. Note reduced embryo size in earlier developmental stages, and deficient head formation, particularly anterior to the pharyngeal arches, across all embryonic stages. E9.5-E10.5, arrowheads indicate vesicle structures of the brain. E9.5, red arrowheads indicate prosencephalon, yellow arrowheads indicate mesencephalon, cyan arrowheads indicate rhombencephalon. E10.5, magenta arrowheads indicate telencephalon, orange arrowheads indicate diencephalon, yellow arrowheads indicate mesencephalon, green arrowheads indicate metencephalon, and purple arrowheads indicate myelencephalon. Note the absence of gross abnormalities in the body plan of the embryo. Putative embryo head faces left. Scale bars: 0.25 mm. See Table S1 for animal numbers.

To explore potential defects in embryonic development caused by the 17q12Del mutation, we examined B6 17q12Del mice during embryogenesis. We concentrated on this background to characterize more severe developmental defects associated with the 17q12Del mutation, as we hypothesized that the low number of live B6 offspring recovered would translate to more overt and consistent abnormalities during development. We examined embryo morphology through a critical window of head development, from E8.5 to E12.5, and observed a range of head and brain anomalies in B6 17q12Del embryos (Fig. 3). This period spans the formation of the neural tube in mid-neurulation (Copp et al., 2003), regionalization of the forebrain (Shimamura and Rubenstein, 1997) and the beginning of neural crest formation (Minoux and Rijli, 2010), to early corticogenesis (Harrison-Uy and Pleasure, 2012; Toma and Hanashima, 2015) and the merging of the major facial prominences (Chai and Maxson, 2006; Feng et al., 2009). We present representative male and female B6 17q12Del embryos at time points and Theiler stages (Richardson et al., 2014; Theiler, 2013) within this window, demonstrating mild (m) or severe (s) abnormalities. See Table S2 for phenotypes associated with mild or severe abnormalities. Although there is a spectrum of abnormal phenotypes represented among these embryos, a few consistencies emerge. In earlier embryonic stages (E8.5-E10.5), B6 17q12Del embryos were smaller than B6 WT, but this difference mostly resolved by E12.5, particularly in mild embryos. Moreover, all B6 17q12Del embryos had deficient head structure development. At E8.5, all anterior structures were disproportionately reduced in size relative to the rest of the embryo, but, by E9.5, the prosencephalon (Fig. 3, red arrowheads) and mesencephalon (Fig. 3, yellow arrowheads) were more clearly affected, whereas the rhombencephalon (Fig. 3, cyan arrowheads) began to develop more normally. This trend continued to E10.5, as in all embryos, telencephalon (Fig. 3, magenta arrowheads) and diencephalon (Fig. 3, orange arrowheads) formation were clearly impaired, whereas the mesencephalon (Fig. 3, yellow arrowheads) appeared affected to varying degrees depending on the severity of the overall phenotype. In contrast, hindbrain structures appeared mostly preserved. The invagination of the midbrain–hindbrain isthmic organizer, indicating the anterior metencephalon (Fig. 3, green arrowheads), was visible in all embryos, although was less distinct in male and female severe embryos. Further, myelencephalon structures (Fig. 3, purple arrowheads) were distinguishable in all embryos. As the embryo continued development to E12.5, the differences between mild and severe abnormalities became more obvious. Mildly anomalous embryos had abnormal or abrogated partitioning or maturation of the brain vesicles, but a discernible head structure continued to form. In contrast, in the more severe deletion embryos, the putative head became more dramatically unstructured, and vesicle distinctions were unidentifiable. We did not observe any consistent difference in severity between male and female embryos.

#### Early newborn development

**Fig. 4.**
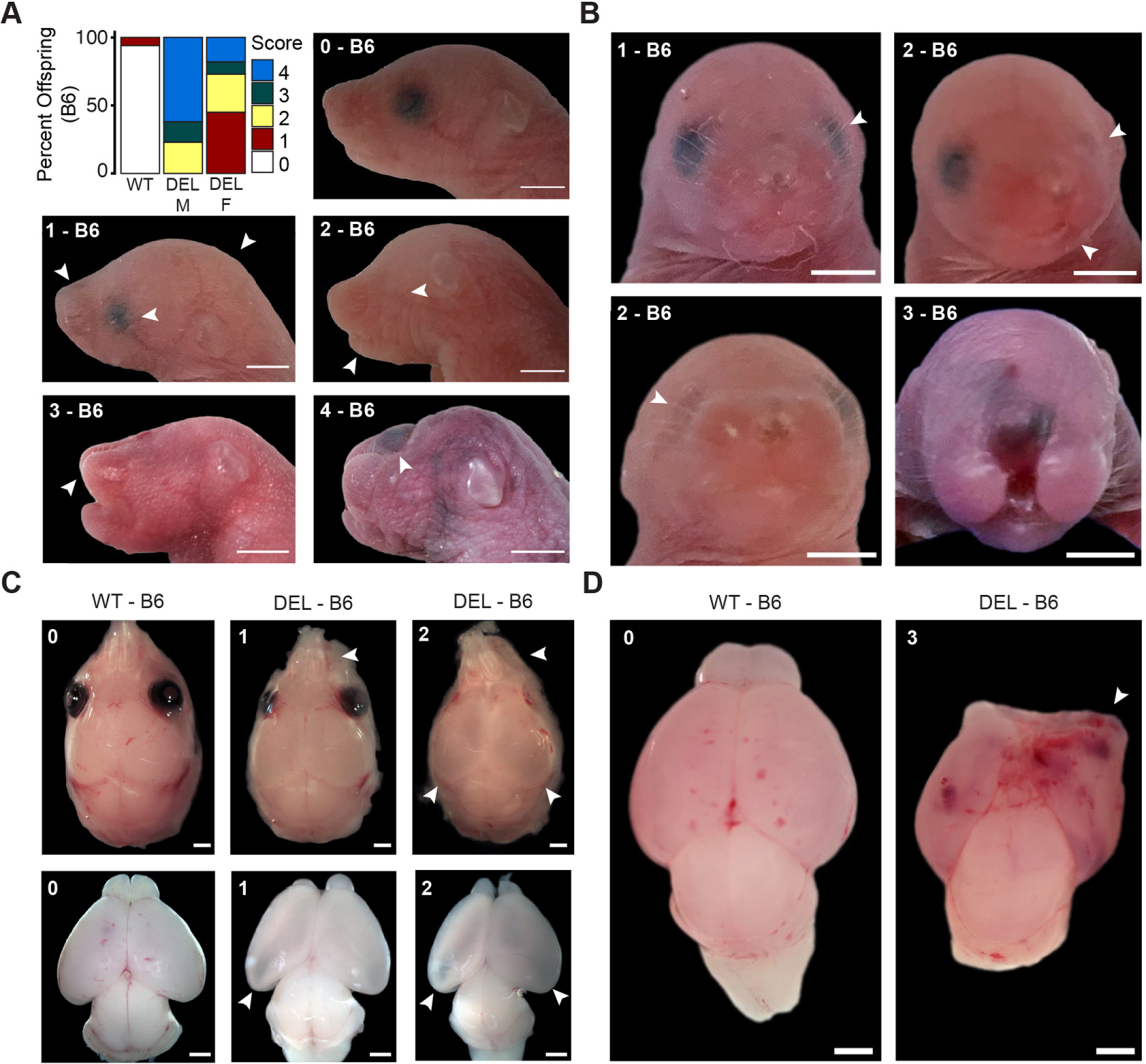
**Neonatal B6 17q12Del mice have abnormal head phenotypes.** (A) Head phenotypes of recovered P0 B6 17q12Del (DEL) and WT offspring. Top left: observed frequency of dysmorphic features in neonatal pups, scored by severity (0-4). Note that more severe scores may include features from milder scores. 0-4, representative pups with designated scores. See Materials and Methods and Table S2 for additional details on scoring. 0, no abnormalities; 1, mild abnormalities (white arrowheads indicate shortened snout, malformed eye and domed skull); 2, moderate abnormalities (white arrowheads indicate absent eye and asymmetric lower jaw); 3, severe abnormalities (white arrowhead indicates incomplete closure of the maxilla); 4, very severe abnormalities (white arrowhead indicates flattened head and hematoma protrusion, indicating severe malformation of brain structures). (B) Representative *en face* images of B6 DEL pups with severity scores 1, 2 and 3. Top left: white arrowhead indicates immature eye structure. Top right: white arrowheads indicate misaligned nose and jaw and absent left eye. Bottom left: white arrowhead indicates absent right eye. (C) Representative crania (top row) and brains (bottom row) from mild and moderate severity B6 DEL offspring compared to WT offspring. DEL(1), white arrowheads indicate shortened nasal bones and hollow cortical structures, indicating deficient hippocampal genesis. DEL(2), white arrowheads indicate asymmetric nasal bones and olfactory bulbs, and hollow cortical structures. (D) Representative brains recovered from B6 WT and severity score 3 B6 DEL pups. Note the profound lack of structure in the anterior right hemisphere (white arrow) and reduced cortical size in the DEL brain. Scale bars: 2.5 mm (A,B); 1 mm (C,D). See Table S1 for animal numbers.

For newborn animals (P0), we quantified the different head and brain malformations observed in newborn B6 17q12Del offspring (Fig. 4A). We scored these phenotypes from 0 to 4 in order of increasing severity (0, no abnormalities; 1, mild abnormalities; 2, moderate abnormalities; 3, severe abnormalities; 4, very severe abnormalities; see Fig. 4 legend, Materials and Methods and Table S2 for details). Although we did not observe a difference in the proportion of surviving male versus female B6 17q12Del offspring (Fig. 2B), we noted that male B6 17q12Del pups more frequently had severe phenotypes, whereas female B6 17q12Del pups were more frequently mild or moderate (Fig. 4A). In Fig. 4, white arrowheads indicate key features of select B6 17q12Del offspring from the severity categories, including the following: (1) shortened snout, domed head and unilateral mild hypoplasia of the eye; (2) asymmetry of the mandible/maxilla and unilateral severe hypoplasia of the eye; (3) incomplete closure of the maxilla; and (4) severe malformations of the head. *En face* images of select B6 17q12Del offspring highlighting facial asymmetry are shown in Fig. 4B, in which white arrowheads indicate unilateral mild eye hypoplasia (1), unilateral severe eye hypoplasia and asymmetry of the nasal bones/misalignment with the mandible (2, top right) and unilateral severe eye hypoplasia (2, bottom left). An example of lack of mandibular fusion is also shown (3). We dissected the brains and skulls of animals that were in the severity score category of 1 or 2 (Fig. 4A) to further examine neural and cranial malformations underlying the domed head appearance (Fig. 4C). We first observed that abnormalities in the pup snout were driven by malformations of the nasal bones; in Fig. 4C (top row), white arrowheads indicate shortened bones relative to WT (1), and nasal bones angled away from the midline and asymmetrical olfactory bulbs (2). Further, we observed that the domed head was frequently accompanied by anomalies in brain structure, visible as apparently hollow regions (Fig. 4C, top and bottom rows, white arrowheads at the caudal brain), suggestive of cortical thinning and deficient formation of the hippocampus, evident to different degrees of severity. Dissection of a recovered brain structure from an offspring with a severity score of 3 revealed profound malformations and asymmetric underdevelopment of the cortex (Fig. 4D), including disrupted cortical hemispheres and abnormal vascularization.

To confirm the structural underpinnings of the hollow regions of the B6 17q12Del brains, we examined Nissl-stained coronal sections of representative P0 brains from B6 17q12Del pups scored 1 and 2, both female (Fig. S1). Comparing matched sections revealed few overt abnormalities in the 1-scored brain. In contrast, the 2-scored brain had extreme ventricular dilatation, cortical thinning and hippocampal hypoplasia, whereas diencephalon and midbrain structures appeared less affected.

### Increased severity of craniofacial and brain morphology abnormalities in 17q12Del adult males in CD-1 background

#### Craniofacial defects

**Fig. 5.**
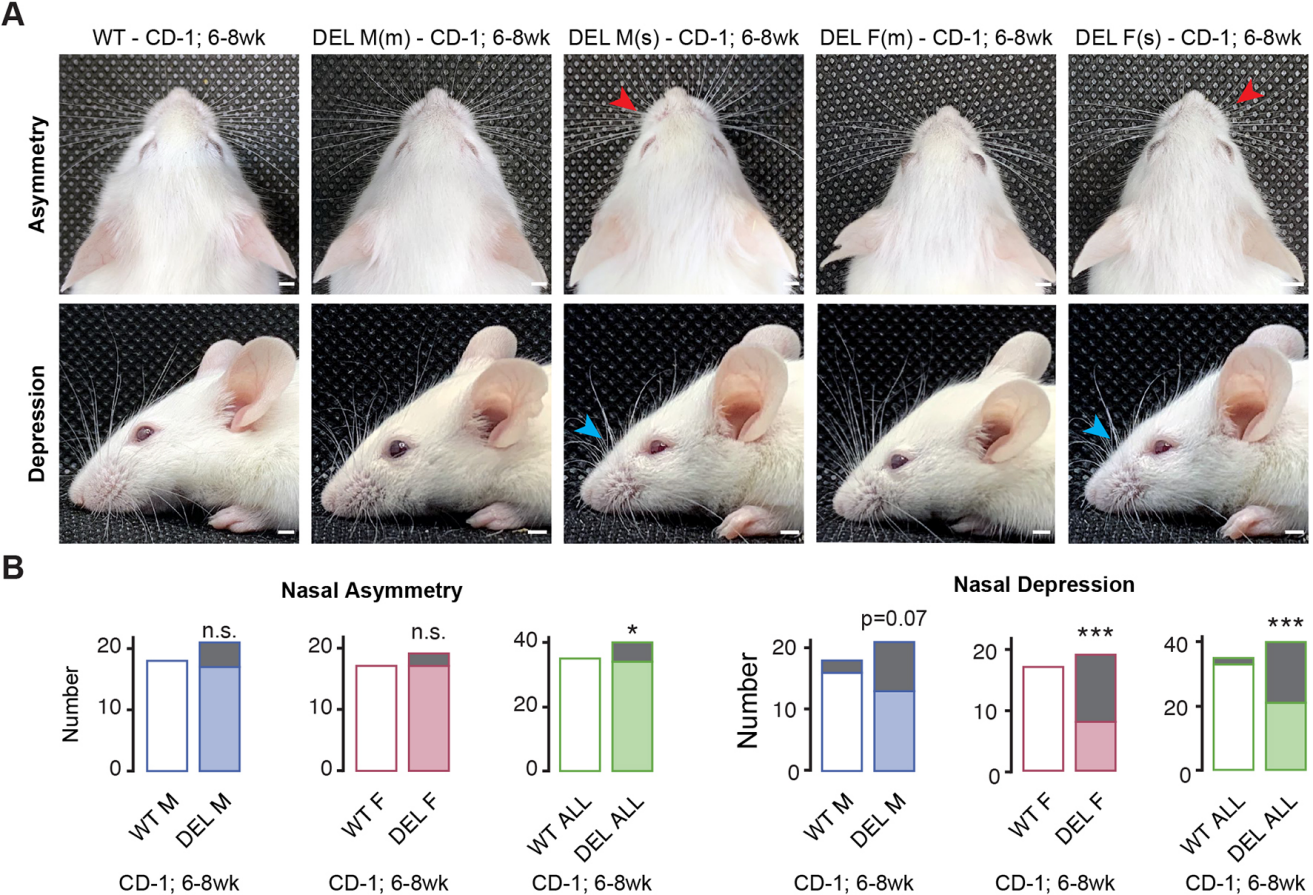
**Frontonasal abnormalities of CD-1 17q12Del mice.** (A) Representative images of mild (m) and severe (s) male and female CD-1 17q12Del (DEL) mice with and without characteristic malformations of the nose compared to CD-1 WT. Top row: overhead view. Red arrows indicate left or right asymmetry of the nasal bones. Bottom row: side view. Cyan arrowheads indicate depressions of the nasal bridge. Mice in top and bottom images are separate individuals. Scale bars: 2.5 mm. (B) Number of male (left), female (center) and all (right) CD-1 WT and DEL mice with nasal asymmetry (left) or nasal depression (right). Gray regions indicate number of animals with abnormalities. See Table S1 for animal numbers. Fisher's exact test for significance. *P<0.05; ***P<0.001; n.s., not significant.

As CD-1 17q12Del mice survived to adulthood, we concentrated our subsequent studies on these mice to interrogate phenotypes associated with the human disorder. We hypothesized that CD-1 17q12Del mice show craniofacial and brain defects, as 17q12Del patients frequently have facial dysmorphologies and intellectual disability (Moreno-De-Luca et al., 2010). Phenotypes on the CD-1 background may potentially represent a milder end of a continuum from the severe embryonic and perinatal defects seen with increasing B6 background. We examined 6- to 8-week-old 17q12Del mice on the CD-1 background to characterize overt craniofacial malformations in adult mice. In a subset of CD-1 17q12Del mice, we observed a pattern of abnormalities of the nasal bones, primarily falling into either lateral asymmetry or depression of the nasal bridge (Fig. 5A). We grouped CD-1 17q12Del mice without nasal abnormalities as mild (m) phenotype and those with either lateral asymmetry (Fig. 5A, red arrowheads) or depression of the nasal ridge (Fig. 5A, cyan arrowheads) as severe (s) phenotype. We observed these abnormalities in both male and female CD-1 17q12Del mice and did not observe a consistent trend in the direction (left or right) of the nasal asymmetry. Quantification (Fig. 5B) revealed that the incidence of nasal asymmetry was low and statistically insignificant when males and females were considered separately [males, 95% confidence interval (CI): 0.598-infinity (Inf), *P*=0.11; females, 95% CI:0.169-Inf, *P*=0.487], but significant when all CD-1 WT mice were compared against all CD-1 17q12Del mice (95% CI: 1.10-Inf, *P*=0.027). We observed indentation of the nasal bridge in a larger proportion of CD-1 17q12Del mice than CD-1 WT mice. This frequency approached significance in males (95% CI: 0.758-53.32, *P*=0.074), but was highly significant in females (95% CI: 3.852-Inf, *P*=0.00014) and when both sexes were combined (95% CI: 2.993-140.6, *P*=6.24×10^−5^). Interestingly, nasal asymmetry was more frequent in males, whereas depression of the nasal ridge was more frequent in females in this group, but this observation should be validated in a larger population.

#### Postnatal brain morphometry

To identify brain morphological abnormalities, we selected representative mild and severe 6- to 8-week-old male and female CD-1 17q12Del mutants, and Nissl-stained six coronal sections to compare against CD-1 WT. CD-1 17q12Del brains annotated as mild had no nasal abnormalities, whereas those annotated as severe had either nasal bone asymmetry or depression of the nasal ridge (see Fig. 5). Using six coronal sections from anterior to posterior regions of the brain, we observed abnormalities in CD-1 17q12Del mice on a spectrum of severity. In both male CD-1 17q12Del mice (Fig. S2), we noted lateral ventricular dilatation in the mild and severe male mice relative to the WT mouse, which was much more prominent in the severe mouse. In the severe mouse, lateral ventricular dilatation was accompanied by thinning of the corpus callosum and the posterior cortex. In contrast, although we observed similar abnormalities in female CD-1 17q12Del mice (Fig. S3), the observed phenotypes were less severe. The female mild mouse had few structural abnormalities. In the female severe mouse, we observed asymmetric lateral ventricle dilatation, which was also accompanied by asymmetric posterior cortical thinning.

#### Small animal MRI analysis

**Fig. 6.**
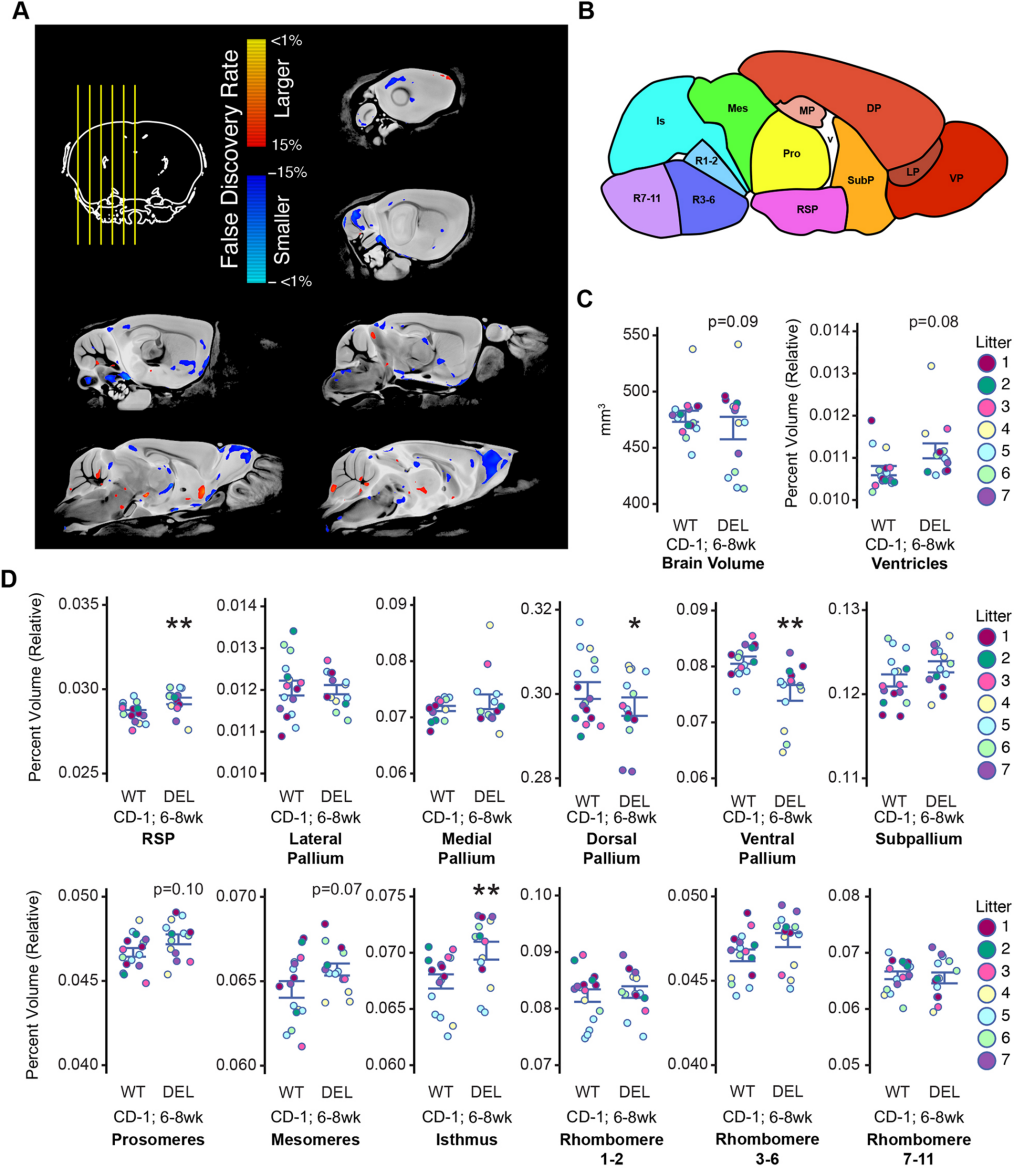
**Magnetic resonance imaging (MRI) reveals brain volume alterations in CD-1 17q12Del mice.** (A) Representative serial sagittal MRI images of CD-1 17q12Del mice (DEL) compared to CD-1 WT. Top left: yellow lines overlaid on coronal section indicate position of sagittal sections. Red (positive) and blue (negative) indicate false discovery rates (FDRs) of regions larger and smaller (respectively) by absolute volume. (B) Representative sagittal section reflecting developmental structural ontogeny bins used for classifying MRI volumes. DP, dorsal pallium; Is, isthmus; LP, lateral pallium; Mes, mesomeres; MP, medial pallium; Pro, prosomeres; RSP, rostral secondary prosencephalon; R1-2; rhombomeres 1-2; R3-6; rhombomeres 3-6; R7-11; rhombomeres 7-11; SubP, subpallium; VP, ventral pallium. Based on Allen Developing Mouse Brain Atlas (https://atlas.brain-map.org/atlas?atlas=181276165#atlas=181276165&plate=100883770). (C) The DEL brain volume is smaller than that of WT (left), whereas relative ventricle volume is higher (right), approaching statistical significance. (D) DEL relative brain region volumes. (C,D) Point color reflects litter. See Table S1 for animal numbers. (A,C,D) Mixed effects model for genotype with litter as a random effect, followed by FDR correction for multiple comparisons. Error bars reflect s.e.m. for each group. *FDR<0.05; **FDR<0.01.

For a further assessment of 17q12Del brain structural abnormalities, we performed magnetic resonance imaging (MRI) on 6- to 8-week-old CD-1 male WT and 17q12Del mice, as we had observed that male CD-1 17q12Del mice had more severe abnormalities. We reasoned that the outbred nature of the CD-1 background approaches the variability observed in human populations (Aldinger et al., 2009). To account for the statistical variability, we used mixed effects models to incorporate litter as a random effect (Golub and Sobin, 2020) (see Materials and Methods for details). We identified 182 brain regions and compared them between male CD-1 WT and 17q12Del mice, using this mixed effects model, followed by a false discovery rate (FDR) correction for multiple comparisons (Genovese et al., 2002) [see Table S3 for volumes, percentage differences, *t*-statistics, *P*-values and FDR values; FDR values below 0.05 (5%) were considered significant]. Fig. 6A shows six sagittal MRI sections, with overlaid aggregated absolute volume differences from CD-1 17q12Del mice at a 15% FDR. When considering absolute volumes, we found that the five layers of the olfactory bulb (glomerular, external plexiform, mitral cell, internal plexiform, granule cell) were significantly smaller in the CD-1 17q12Del mice than in WT mice, by an average of 15% (Table S3). The lateral olfactory tract was also reduced by 9%. We detected significant reductions in the cerebellar crus 1 of the ansiform lobule (6%) and the primary auditory cortex (6.3%). However, we observed that CD-1 17q12Del brain volume trended lower than that of WT [*t*(22.84)=1.77, *P*=0.0896, 2.2% difference] (Fig. 6C, left), and subsequently only considered brain region volumes normalized to total volume, or relative volume. Controlling for this variation revealed significant increases in some brain regions as well as decreases, including a 7% increase in the cerebellar fastigial nucleus, a 3.5% increase in the hypothalamus, and a 6.7% and 8.3% increase in the medial and lateral septa, respectively (Table S4).

Considering our observation of gross abnormalities from the B6 17q12Del embryos and newborns, we interrogated alterations in brain region volume from the perspective of brain development. We binned the 182 regions, using relative volumes, into 12 developmental areas and the ventricular space, guided by structural ontogeny information available from the Allen Developing Mouse Brain Atlas (https://atlas.brain-map.org/atlas?atlas=181276165#atlas=181276165&plate=100883770) (Fig. 6B) (see Table S5 for a list of regions and developmental bins). Using these bins and the same statistical procedure, we identified significant differences in several areas (Fig. 6D). In the 17q12Del mouse, the dorsal pallium [*t*(22.374)=3.007, *q*=0.021], comprising the majority of the cerebral cortex, and ventral pallium [*t*(22.86)=4.084, *q*=0.003], comprising the olfactory bulb, endopiriform and piriform cortex, were significantly reduced by 1.3% and 7.2%, respectively. In contrast, the rostral secondary prosencephalon (RSP) [*t*(23.25)=−3.57, *q*=0.007], which includes the hypothalamus and mammillary bodies, and isthmus [*t*(22.88)=−4.405, *q*=0.003], which includes the cerebellum, were significantly increased by 2.3% and 4%, respectively. In addition, the prosomere [*t*(28)=−1.991, *q*=0.104] (thalamus, periaqueductal grey), mesomere [*t*(23.63)=−2.346, *q*=0.072] (midbrain, inferior colliculus, superior colliculus) and ventricular [*t*(22.96)=−2.207, *q*=0.081] (Fig. 6C, right) region volumes were increased (1.6%, 1.8% and 4.4%, respectively), approaching statistical significance (see Table S6).

### 17q12Del mice demonstrate renal and diabetes phenotypes with sex-specific penetrance

#### Animal growth

**Fig. 7.**
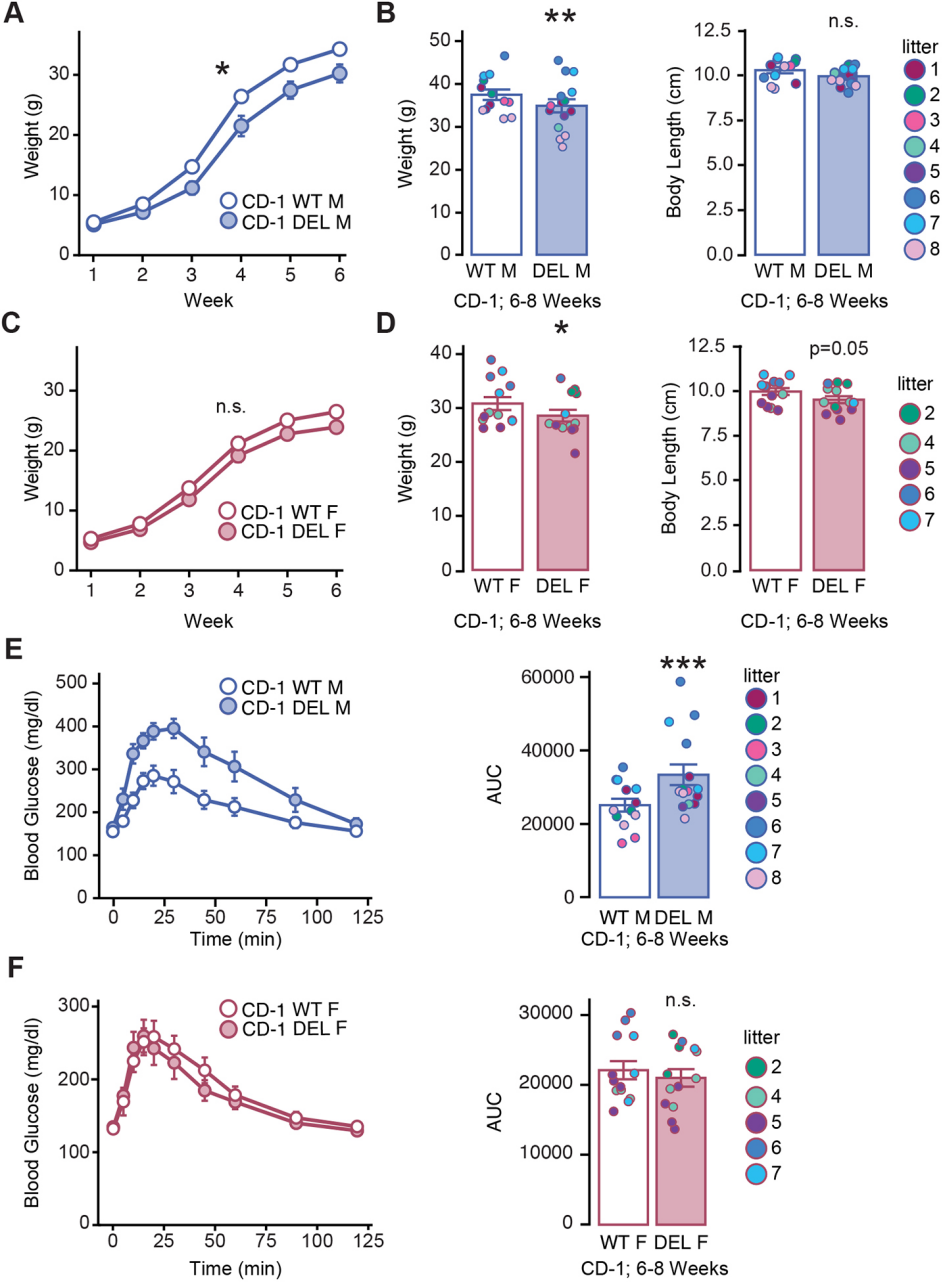
**The 17q12Del mutation causes sex-specific effects on body weight and glucose metabolism.** (A) Growth curves of male CD-1 WT and 17q12Del (DEL) mice from week 1 to week 6 of age. The growth curve of male CD-1 DEL mice diverges from that of male WT mice between weeks 2 and 3, and the effect of genotype on weight over time is significant. (B) At 6-8 weeks, male CD-1 DEL mice weigh significantly less than male WT littermates, but their body length is not significantly different from that of male WT mice. (C) Growth curves of female CD-1 WT and DEL mice from week 1 to week 6 of age. The growth curve of female CD-1 DEL mice diverges from that of female WT littermates by week 6, but the effect of genotype on weight is not significant. (D) At 6-8 weeks, female CD-1 DEL mice weigh significantly less and are significantly smaller than female WT littermates. (E) Intraperitoneal glucose tolerance test (IPGTT) of male CD-1 DEL mice and WT littermates. Left: IPGTT line graph of male CD-1 WT and DEL mice, reflecting blood glucose levels versus time. Right: area under the curve (AUC) of blood glucose from the IPGTT time curve. The AUC of male CD-1 DEL mice is significantly elevated compared to that of male WT mice. (F) IPGTT of female CD-1 DEL mice and WT littermates. Left: IPGTT line graph of female CD-1 WT and DEL mice. Right: AUC of blood glucose from the IPGTT time curve. See Table S1 for animal numbers. (B,D-F) Point color reflects litter. (A,C) Mixed effects model for interaction of genotype and week with male (A) or female (C) mouse as a random effect. (B,D) Mixed effects model for genotype with litter as a random effect. (E,F) Mixed effects model for genotype, with weight as a fixed effect and litter as a random effect. Error bars reflect s.e.m. for each group or time point. *P<0.05; **P<0.01, ***P<0.001; n.s., not significant.

Humans with 17q12Del disorder frequently present with RCAD, with variable expressivity and incomplete penetrance, encompassing a spectrum of renal abnormalities and MODY5. Less frequently, 17q12Del syndrome patients also have short stature (Mitchel et al., 2016). To examine these phenotypes in the 17q12Del mouse model, we studied 6- to 8-week-old male and female CD-1 mice, using mixed models to incorporate litter as a random effect. We observed a decrease in CD-1 17q12Del growth, weight and body length, with differential expression between males and females. We first evaluated the growth rate of male CD-1 17q12Del mice over the course of 6[Supplementary-material sup1]weeks. Genotype had a significant interaction with weight over time in male mice [*t*(33.865)=2.188, *P*=0.036] (Fig. 7A). We confirmed this observation by finding that 6- to 8-week-old male mice weighed less than their WT littermates [*t*(21.02)=3.631, *P*=0.00156], although their body lengths were not significantly different [*t*(23.02)=1.544, *P*=0.136] (Fig. 7B). In contrast, there was no significant relationship between genotype and the time course of weight gain in female mice [*t*(23.39)=1.147, *P*=0.263] (Fig. 7C). However, in 6- to 8-week-old female mice (from the same litters as those in Fig. 7B), we found that female CD-1 17q12Del mice weighed less than female CD-1 WT littermates [*t*(21.87)=2.329, *P*=0.0295] and had reduced body length [*t*(23.18)=2.056, *P*=0.0512] (Fig. 7D).

#### Glucose homeostasis

To evaluate diabetic phenotypes in CD-1 17q12Del mice, we performed an intraperitoneal glucose tolerance test (IPGTT) on 6- to 8-week-old mice. We observed that male CD-1 17q12Del mice had impaired blood glucose clearance relative to CD-1 WT males (Fig. 7E), but there was no difference in blood glucose clearance between CD-1 17q12Del and CD-1 WT females (Fig. 7F). To quantify these differences, we calculated the area under the curve (AUC) for each mouse. Because CD-1 17q12Del males and females weighed significantly less than WT littermates, and because we determined that weight was highly correlated with AUC in both male [Pearson's correlation coefficient (PCC)=0.551, *t*=3.368, *P*=0.0024] and female (PCC=0.755, *t*=5.641, *P*=8.289×10^−6^) mice, we added weight as a fixed effect to the mixed effects model to compare AUC. Male CD-1 17q12Del AUC was elevated compared to that of male CD-1 WT [*t*(27.26)=−5.085, *P*=2.036×10^−5^) (Fig. 7E, right), but, remarkably, there was no difference between female CD-1 17q12Del AUC and that of female CD-1 WT [*t*(26)=−0.666, *P*=0.511] (Fig. 7F, right). We examined the pancreas of these animals using Hematoxylin and Eosin (H&E) staining and did not find any abnormalities.

#### Kidney phenotypes

**Fig. 8.**
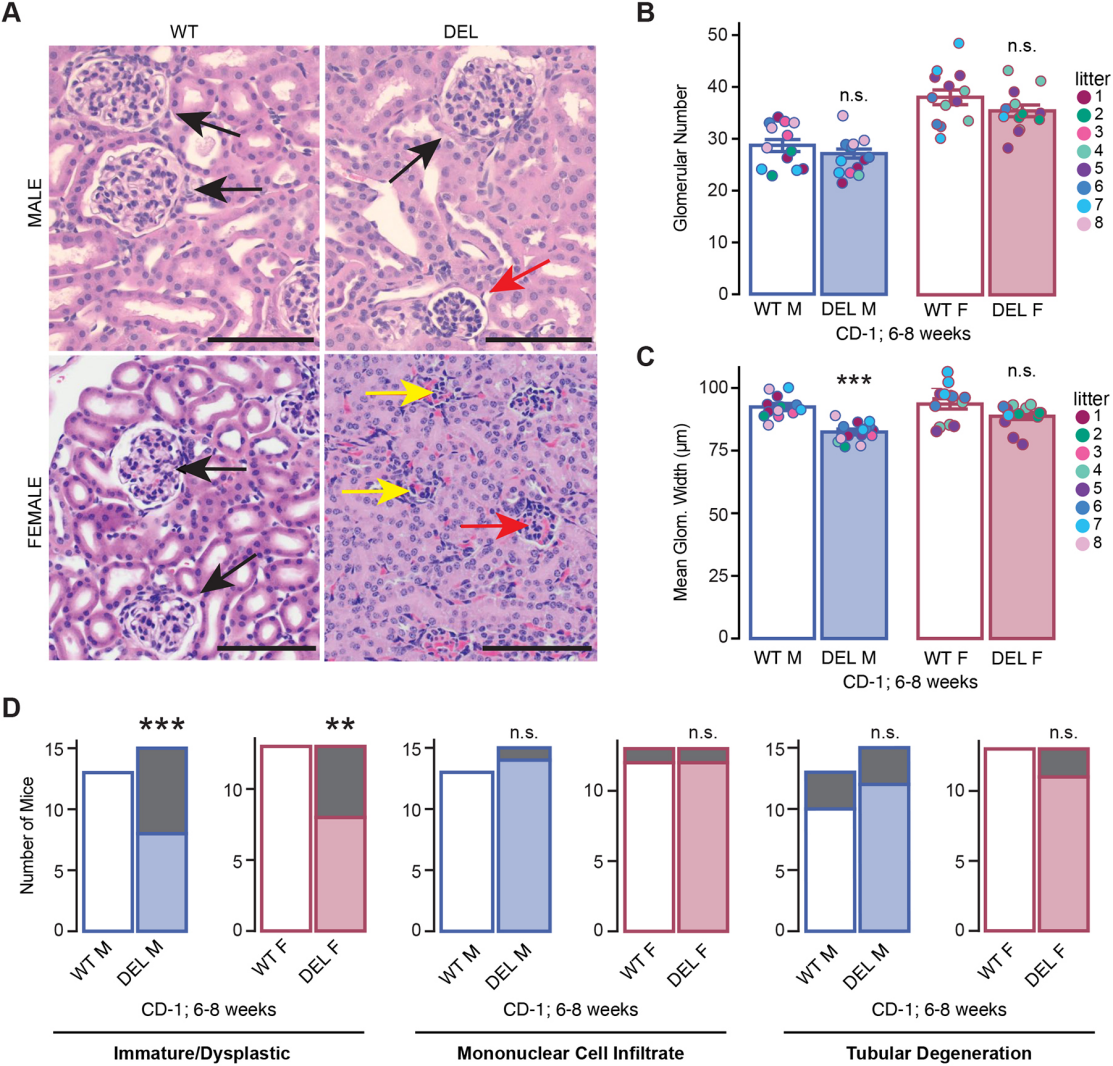
**17q12Del mutation causes renal abnormalities.** (A) Representative H&E staining of kidney sections from 6-week-old CD-1 WT and 17q12Del (DEL) male (top row) and female (bottom row) mice. Black arrows indicate normal glomeruli, red arrows indicate immature glomeruli, yellow arrows indicate dysplastic glomeruli. Scale bars: 100 µm. (B) Mean glomerular number is reduced, but not significantly, in CD-1 DEL male and female mice. (C) Mean glomerular width is significantly reduced in male, but not female, CD-1 DEL mice. (B,C) Mixed effects model for genotype tested against the null model, with weight as a fixed effect and litter as a random effect. (D) CD-1 WT and DEL mice with immature and/or dysplastic glomeruli, mononuclear cell infiltrate or tubular degeneration in male and female mice as a proportion of total animals examined. Gray indicates numbers of animals with the indicated anomalies. See Table S1 for animal numbers. (B,C) Point color reflects litter. Fisher's exact test for significance. Error bars reflect s.e.m. for each group. **P<0.01, ***P<0.001; n.s., not significant.

In the same group of mice, we investigated the renal phenotypes of the CD-1 17q12Del mice to determine the incidence of any abnormalities. We directly assessed renal pathology by performing H&E staining on renal sections from CD-1 male and female 17q12Del mice (Fig. 8A). The mean number of glomeruli was reduced in both male (WT, 28.7±1.15; 17q12Del, 27.1±0.867) and female (WT, 37.9±1.43; 17q12Del 35.3±1.12) CD-1 17q12Del mice, although this difference was not significant in either group [with weight as fixed effect and litter as random effect; male *t*(24.72)=1.164, *P*=0.255; female *t*(23)=1.82, *P*=0.082] (Fig. 8B). Further, male CD-1 17q12Del mice had a consistently smaller mean glomerular diameter than that of male WT littermates [WT, 92.2±1.19 µm; 17q12Del, 82.3±0.933; *t*(24.93)=6.214, *P*=1.7×10^−6^], but this effect was modest and not significant in female CD-1 17q12Del mice [WT, 93.8±2.05 µm; 17q12Del, 88.9±1.35 µm; *t*(22.99)=1.356, *P*=0.188] (Fig. 8C). In several male and female CD-1 17q12Del mice, we observed immature and/or dysplastic glomeruli (Fig. 8A). Although we did not observe gross kidney malformations, these microscopic defects are highly suggestive of abnormalities in renal development. These effects demonstrated incomplete penetrance. The presence of immature and/or dysplastic glomeruli was enriched in the CD-1 17q12Del group in both male (95% CI: 1.69-Inf, *P*=0.0069) and female (95% CI: 1.11-Inf, *P*=0.039) mice by Fisher's exact test (Fig. 8D, top). We also observed an incidental number of 17q12Del mice with other renal anomalies, including tubular degeneration (Fig. 8D, middle) and mononuclear cell infiltrate (Fig. 8D, bottom), but the frequencies of these observations were comparable with those in WT animals. We also evaluated the liver and female reproductive tract, which we hypothesized to be affected by the haploinsufficiency of the 17q12Del genes. We observed no abnormalities in the reproductive tract, and although microvesicular hepatocellular vacuolation, characteristic of lipid accumulation, was detected in a subset of animals, this finding was detected equally in CD-1 WT and CD-1 17q12Del males and females.

## DISCUSSION

In this study, we established a mouse model of 17q12Del syndrome, a rare CNV disorder associated with both neuropsychiatric disorders and medical syndromes. By propagating the 17q12Del mutation onto both the inbred B6 and the outbred CD-1 genetic backgrounds, we explored phenotypes associated with the 17q12Del through a range of penetrance and expressivity. The B6 background confers high penetrance to the 17q12Del, and the mutation is non-viable on an isogenic B6 background. With a majority B6 background, 17q12Del offspring have profound and generally lethal abnormalities in embryonic head and brain development. By contrast, the 17q12Del appears to have a lower penetrance and milder phenotype on the CD-1 background, and animals live into adulthood. It is important to note that we did not perform a comprehensive study of CD-1 embryos or neonates, and it is likely that, as a result, we have underestimated the penetrance of 17q12Del on this background. Nonetheless, our study clearly suggests a greater phenotypic severity on the B6 than the CD-1 background.

There are many potential sources for the variation in penetrance and phenotypic severity of the 17q12Del mutation on the B6 and the CD-1 backgrounds. CD-1 mice have larger litter sizes (Silver, 1995; Tanaka, 1998), and have lower rates of pup mortality than B6 mice (Morello et al., 2020; Wright and Brown, 2000), which may create a more permissive environment for 17q12Del mutant survival. In other experimental models, the B6 background has been shown to have a higher penetrance of craniofacial and neurological abnormalities (Cobolli Gigli et al., 2016; Yan et al., 2007), while the CD-1 background is more susceptible to renal injury (Srivastava et al., 2018; Sugimoto et al., 2007), suggesting there are likely strain-dependent influences on the abnormalities we have identified in the 17q12Del model. Further, embryological brain and skull morphologies are significantly different between B6 and CD-1 pups, adding a further possibility that there is enough difference in the developmental trajectory of the two backgrounds to increase or reduce penetrance of the 17q12Del (Motch Perrine et al., 2017). Future studies could consider crossing the 17q12Del onto an inbred background more closely related to CD-1, like FVB/NJ or SWR/J (Aldinger et al., 2009), or onto an outbred background with more genetic diversity, such as the CC or DO strains, to disambiguate some of these possibilities (Svenson et al., 2012; Threadgill et al., 2011).

We used the 17q12Del on the outbred CD-1 background to exploit a background with lower penetrance as well as genetic heterogeneity that more closely resembles the human population to examine 17q12Del phenotypes in adult animals (6-8 weeks). In the kidney, CD-1 17q12Del mice have an increased number of immature and/or dysplastic glomeruli, and male CD-1 17q12Del mice have a reduced mean glomerular width, suggestive of developmental renal abnormalities. Male CD-1 17q12Del mice also have deficient blood glucose clearance, suggestive of abnormalities in glucose metabolism that may presage the development of diabetes, another feature of the human 17q12Del disorder. However, we did not observe any abnormalities of the female reproductive tract. Interestingly, CD-1 17q12Del craniofacial and brain abnormalities may mirror those observed in 17q12Del syndrome patients as well. We observed nasal/facial asymmetry and depression of the nasal bridge in a subset of CD-1 17q12Del mice, features which have been noted in the human disorder (Moreno-De-Luca et al., 2010; Palumbo et al., 2014; Rasmussen et al., 2016). We observed ventricular dilatation and abnormalities of the hippocampus, which are features of some of the few 17q12Del syndrome patients evaluated by MRI (Kasperaviciute et al., 2011; Loirat et al., 2010; Nagamani et al., 2010). Importantly, these features are also frequently found in ASD (Banker et al., 2021; Haar et al., 2016; Turner et al., 2016).

In patients with known biological sex characterized in the DECIPHER database (Firth et al., 2009), more males have been identified with 17q12Del syndrome than females (66 versus 48) as of this writing. Further, the incidence of the primary pathological features associated with 17q12Del syndrome is more frequently observed in males than females. Although there are a spectrum of phenotypes associated with the disorder, many of them can be grouped into five major categories, reflecting the major features of the disorder: facial abnormalities [found in 19/66 (26%) of males, and 9/48 (19%) of females], intellectual disability or developmental delay [37/66 (56%) of males; 21/48 (44%) of females], renal abnormalities [18/66 (27%) of males; 12/48 (25%) of females], diabetes or obesity [3/66 (5%) of males; 5/48 (10%) of females], and brain morphological abnormalities [2/66 (3%) in males; 3/48 (7%) in females]. In the CD-1 17q12Del mouse, we quantified a similar pattern of abnormalities reflecting these features, expressed at a somewhat higher penetrance than in the human disorder. For our study, we counted abnormalities as present or absent (facial, renal), or if they were more than two standard deviations away from the WT mean (glucose homeostasis). We observed, for example, facial abnormalities [10/21 (48%) of males; 13/19 (65%) of females], immature glomeruli [8/15 (53%) of males; 5/13 (38%) of females] and abnormal glucose homeostasis [4/15 (27%) of males; 0/13 (0%) of females]. Importantly, although our data suggest a higher rate of penetrance in our mouse model relative to the human population, characterization of phenotypes in patients is limited.

We observed a spectrum of craniofacial and brain abnormalities in 17q12Del mice. Generally, in our characterization of low penetrance CD-1 offspring, this includes mild nasal dysmorphologies, but higher-penetrance majority B6 offspring have abnormalities that include profound ventricular dilatation, hippocampal hypoplasia and, at the most severe, incomplete closure of the neural tube and maxilla and disruption of all forebrain and midbrain structures. We determined that abnormalities in B6 17q12Del embryos are evident as early as E8.5, suggesting that early developmental events drive many of the brain and craniofacial abnormalities observed in later development. Our MRI study of CD-1 17q12Del male mice revealed a relative reduction in the volume of anterior structures such as the ventral and dorsal pallium, coupled with a relative increase in other more caudal regions including the RSP, prosomeres and mesomeres, and isthmus, while the hindbrain is spared. Although these abnormalities are milder than those in the B6 background, the abnormalities in rostral regions seem common to mice on both backgrounds, suggesting that the 17q12Del mouse brain phenotype is characterized by early developmental patterning defects that predominantly affect anterior structures.

Our findings of strong anterior neural tube (i.e. head and brain) malformation phenotypes in the 17q12Del mouse model provide some insight into the likely genetic underpinnings of the disorder. Although 17q12Del syndrome is caused by haploinsufficiency of 15 protein-coding genes, we hypothesize that *LHX1* has a strong functional link to the pathophysiology of 17q12Del syndrome, as head phenotypes have been robustly demonstrated through previous mouse models exploring *Lhx1* function. *Lhx1* is transiently expressed in the anterior visceral endoderm and axial mesendoderm during early embryonic development, and encodes a transcription factor that acts as an essential head organizer for the developing animal. LHX1 mediates anterior and head patterning, as homozygous null embryos do not form anterior structures (Shawlot and Behringer, 1995), and has been positioned as a hub of a gene regulatory network mediating multiple signaling pathways (McMahon et al., 2019). These signaling pathways include the regulation of Wnt signaling, which LHX1 mediates most directly through the anterior expression of Wnt antagonists CER1 and DKK1 (Fossat et al., 2015). Establishing an appropriate anterior–posterior gradient of Wnt expression is one of the fundamental components of head development (Yamaguchi, 2001), and loci within Wnt pathway components have frequently emerged as risk factors in a number of NDDs, including ASD and schizophrenia (Mulligan and Cheyette, 2017). However, mice heterozygous for *Lhx1* are largely phenotypically normal (Fossat et al., 2015; Shawlot and Behringer, 1995), suggesting that, in isolation, it is unlikely to be haploinsufficient for head development. Consequently, we propose that *Lhx1* haploinsufficiency, although likely the primary driver for the head phenotypes observed in our mouse model, requires the combinatorial haploinsufficiency of some or all of the other genes within the 17q12 locus. Analysis of 17q12Del patient blood samples suggests that the deletion exerts profound alterations of DNA methylation sites, further supporting the possibility of strong combinatorial *cis*-acting regulation of the genes within the interval (Clissold et al., 2018).

Haploinsufficiency of *HNF1B* is presumably the driver for renal abnormalities in both the human disorder and in our mouse model (Bellanné-Chantelot et al., 2004; Bingham et al., 2001; Edghill et al., 2006). Interestingly, in *Xenopus* embryos, there is an *hnf1b* binding site in the *lhx1* promoter, directly regulating nephrogenesis (Drews et al., 2011), which may affect other organ system development. *hnf1b* is transiently expressed in the early developing hindbrain and may have a modifying effect on brain development in combination with other genes (Kim et al., 2005; Wiellette and Sive, 2003). Further, *HNF1B* haploinsufficiency arising from the 17q12Del or point mutations reduces DNA methylation of *NODAL*, a gene crucial for embryonic patterning (Arai et al., 2015; Clissold et al., 2018; Schier, 2009). Homozygous null mutations in zinc finger HIT-type 3 (*ZNHIT3*) and phosphatidylinositol glycan anchor biosynthesis class W (*PIGW*) cause encephalopathy (Anttonen et al., 2017) or intellectual disability (Chiyonobu et al., 2014; Hogrebe et al., 2016), respectively.

Although other genes within the interval have been less comprehensively characterized in humans, animal studies have recently begun to reveal an interesting congruence in the function of a group of genes within the interval. Apoptosis-antagonizing transcription factor (*Aatf*) is essential during embryonic development, early through its involvement in ribosome synthesis (Thomas et al., 2000) and in later stages by mediating neuronal apoptosis (Passananti and Fanciulli, 2007). In contrast, ZNHIT3 is a component in small nucleolar ribonucleoparticle (snoRNP; part of rRNA pre-processing machinery) assembly (Quinternet et al., 2015), as well as a co-receptor for ribophagy with nuclear fragile X mental retardation-interacting protein 1 (NUFIP1) (Wyant et al., 2018). *DDX52*, although itself sparsely characterized, encodes a DEAD-box RNA helicase and is likely also involved in RNA processing (Linder and Jankowsky, 2011). Finally, *Tada2a* encodes the protein more frequently known as ADA2A, which is an adapter protein that forms part of the ATAC histone acetyltransferase complex, the function of which is essential for embryonic and neural tube development (Guelman et al., 2009), and also forms a complex with AATF (Caliskan et al., 2017). Importantly, single-cell RNA-sequencing studies suggest that these five genes are robustly expressed during early embryonic development (Nowotschin et al., 2019), suggesting their likely involvement in early expression and patterning events. These genes may be likely candidates for mediating the expressivity of *Lhx1* haploinsufficiency and should be a focus of future study.

In conclusion, we have established a novel mouse model of 17q12Del syndrome, a CNV disorder linked with intellectual disability, facial dysmorphologies and renal dysfunction. This mouse model has profound head and forebrain development phenotypes on both inbred and outbred genetic backgrounds, providing strong support that the genes within the 17q12 interval are involved in neurodevelopmental processes and providing another example of oligogenic interactions driving the development of NDDs. Study of the 17q12Del mouse will give further insight into the 17q12Del disorder, as well as potentially the etiology of non-syndromic forms of NDDs.

## MATERIALS AND METHODS

### Mouse lines

All experiments involving live animals were conducted in accordance with the National Institutes of Health Guide for the Care and Use of Laboratory Animals (National Research Council of the National Academies, 2011) under a protocol approved by the Brown University Institutional Animal Care and Use Committee. Animal numbers used for each experiment are detailed in Table S1.

#### 17q12 model generation

We constructed the 17q12Del mouse model using CRISPR/Cas9-mediated genome editing (Mouse Transgenic and Gene Targeting Facility at Brown University), as described herein. A 1.2[Supplementary-material sup1]Mb region of the mouse genome syntenic to the 17q12 1.4Mb deletion locus in human was identified. sgRNAs were designed to target regions upstream of *Hnf1b* (GTAGTGCAGTGAGACCCACC, AGTGGTGCCCCTCCCGACAT, TGCACTACCCATGTCGGGAG) and downstream of *Znhit3* (TGCTCTTCCAGAGGAGCCTA, CTGGAGCCAGAGTTACATGT, CCAGCTCCCACATGTA ACTC) to generate breakpoints. A linker (ssDNA) (GAGCTGAGTTACTGGGAAGGGTCAGAGCCCGGTGGGTCTCACTGCACTAC_GTAACTCTGGCTCCAGGGAATCCAGCACCATCATGGTCTCCAAGAGTTCCATACTCA) was additionally introduced to facilitate the joining of the deletion. The deletion was produced in a founder on a C57BL/6N (B6) (RRID:IMSR_JAX:005304) background, between chr11:83791635-84938224 Mb [GRCm38.p6 (mm10), NCBI Reference Sequence, NC_000077.6], matching the consensus human deletion between chr17:31.8-33.2 Mb (GRCh38.p13, NCBI Reference Sequence, NC_000017.11). The presence of the mutation was confirmed by PCR genotyping (Forward, 5′-AGAGCAGCCGATGCTCTTG-3′; Reverse, 5′-GGCAGGTGGATCTCTACGAG-3′) and Sanger sequencing (see Fig. 1). After generating the initial deletion mouse, we bred the founder to a CD-1 (RRID:IMSR_CRL:022) mouse to generate F1 founders, with 50% B6:50% CD-1 background.

#### Embryo and perinatal studies: ≥75% B6

To generate animals for embryological and perinatal studies, we attempted to achieve a majority B6 background with the following strategy. The 50% B6:50% CD-1 background F1 17q12Del males were crossed with 100% B6 WT females to generate 75% B6 17q12Del embryos and pups. For successive generations, 50% B6:50% CD-1 background F1 17q12Del males were crossed with 100% CD-1 WT females to generate 25% B6 17q12Del males. These F2 17q12Del males were crossed with 100% B6 females to yield 62.5% B6 17q12Del offspring, a fraction of which were viable. Then, 62.5% B6 17q12Del males were crossed with 100% B6 WT females to generate 82.5% B6 embryos and pups. We observed that 75% and 82.5% B6 offspring had similar phenotypes and considered them together as ‘≥75% B6’.

#### Postnatal studies: ≥97% CD-1

To generate animals for postnatal studies, we achieved a majority CD-1 background with the following strategy. The 50% B6:50% CD-1 background F1 males were crossed with CD-1 females to generate 75% CD-1 background 17q12Del animals. We continued to outcross 17q12Del animals onto the CD-1 background for a minimum of five generations to achieve ≥97% CD-1 background. As we hypothesized that 17q12Del females have reproductive abnormalities, we used 17q12Del males to breed each generation. As the CD-1 strain is outbred and not isogenic, we considered outcrossing for ≥5 generations to be adequately genetically homogeneous for this study. The number of offspring surviving to weaning in the F5+ generations was considered our total offspring for Mendelian analysis, and a subset of these animals were used for all subsequent experiments. Three (out of 130) 17q12Del CD-1 animals died or were euthanized between weaning and 6-8 weeks of age – one female died of unknown causes, one female was euthanized for malocclusion, and one male was euthanized for hydrocephaly – and were not used for additional experiments.

### ≥75% B6 timed pregnant embryo collection

17q12Del males and B6 females were mated at 18:00, with that point considered E0. E0.5 was set as the subsequent morning. Pregnancies were monitored by daily weighing females (Heyne et al., 2015). At the designated gestational age, pregnant dams were euthanized with 100 mg/kg sodium pentobarbital+12.5 mg/kg sodium phenytoin (Beuthanasia-D solution). Embryos were collected and stage confirmed by morphology. The yolk sac was used for PCR 17q12Del detection and sex determination (SRY Forward, 5′-TCTTAAACTCTGAAGAAGAGAC-3′; SRY Reverse, 5′-GTCTTGCCTGTATGTGATGG-3′).

### ≥75% B6 neonatal pup scoring

Pregnant B6 females were monitored daily until giving birth, upon which pups were collected. Live and dead pups were examined, and abnormalities were documented. When possible, brains were dissected out from skull and stored in 10% phosphate-buffered formalin (Sigma-Aldrich). Internal organs were also examined. Pups were subsequently genotyped. Eight litters were used for this experiment, and a total of 71 pups (47 WT, 24 17q12Del). Pups were binned into five groups, which were assigned an increasing severity score based on craniofacial malformations and brain abnormalities. Note that more severe scores may include features from milder scores. ‘Hollow’ brain observation refers to a phenotype arising from the combination of cortical thinning, ventricular dilatation and hippocampal hypoplasia (see Fig. 5C and Fig. 6). Pups scored as 0 had no observed abnormalities. Pups scored as 1 had abnormalities including shortened snout/nasal bones and/or reduced brain size and/or hollow brain and/or mild eye abnormalities. Pups scored as 2 had a greater number of abnormalities in addition to overt asymmetry of the nose and/or maxilla and profound underdevelopment of one eye. Pups scored as 3 had incomplete closure of the maxilla and/or unilateral malformations of the cortical hemispheres (including protrusions through the skull). Pups scored as 4 had incomplete closure of the maxilla and/or profound brain abnormalities, including lack of differentiation, incomplete closure of the neural tube, protrusions through the skull or lack of all forebrain structures. The number of pups scored as 4 may be inflated due to maternal cannibalism, but pups were carefully inspected to examine remaining cranial structures for malformations. See Table S2 for a summary of scoring criteria.

### aCGH

DNA was extracted from tail lysate from three pairs of WT 17q12Del offspring using standard protocols (DNeasy Blood and Tissue Kit, Qiagen). For this experiment, F2 (25% B6:75% CD-1) animals were used. Whole-genome aCGH was performed using SurePrint G3 Mouse CGH Array Kit (4×180K) (Agilent; CD Genomics). Samples were quality control checked, and WT and deletion samples were hybridized to fluorescent probes and the array. The ADM-2 aberration algorithm was used to detect variations in all three sample sets. A highly significant deletion covering the expected 11qC locus was detected in all three pairs. No other significant aberrations spanning more than one probe were consistently detected in all three pairs. Log2 fluorescence intensity ratios were plotted to visualize the deletion in Fig. 1E.

### ≥97% CD-1 IPGTT

IPGTT was performed following standard protocols (Ayala et al., 2010). Five hours prior to testing, mice were weighed and individually housed,and food was removed. A 20% dextrose (VWR) solution was prepared in 0.9% sterile saline (Fisher Scientific). Five minutes prior to testing, mouse tail tips were clipped, and fasted blood glucose was measured using an Aviva Plus glucometer and test strips (Accu-Chek). At the start of testing, each mouse was injected with a dextrose bolus at 2 g/kg. Blood glucose was measured at intervals of 5, 10, 15, 20, 30, 45, 60, 90 and 120 min.

### ≥97% CD-1 tissue preparation

Mice were anesthetized with Beuthanasia-D solution and transcardially perfused with 1× phosphate-buffered saline (PBS) followed by 10% phosphate-buffered formalin. Left and medial lobes of the liver, kidneys, pancreas and female reproductive tract were removed and stored in 10% phosphate-buffered formalin at 4°C. Brains were removed from skulls, stored in 10% phosphate-buffered formalin for 48 h at 4°C, transferred to 30% phosphate-buffered sucrose, and sectioned or stored at −80°C.

### ≥97% CD-1 histology

Liver, kidney, pancreas and female reproductive tract (uterus, cervix, oviducts) histology was performed. The collected tissues from all mice were processed, embedded in paraffin, sectioned, and stained with H&E. Tissues were scored for microscopic abnormalities. Additional evaluation of the kidney glomeruli was performed, including a calculation of the number of glomeruli per five 20× fields and measurement of glomerular width. The numbers of glomeruli were calculated from evaluation of two fields in the outer cortex near the capsule from the kidney cross-section and three from the longitudinal section. The glomerular width was measured using the line measure tool in the CaptaVision+ software after calibration with a stage micrometer [Calibrated Stage Micrometer KR-851 (1×3), Serial Number 12533]. Ten glomeruli were selected from each animal, four from the cross-section of the kidney and six from the longitudinal section of the kidney. A single measurement was taken across the widest aspect of each selected glomerulus. Immature glomeruli were small in size, composed of densely packed, frequently cuboidal cells and normal in morphology. Dysplastic glomeruli were small in size, malformed or partially formed and sparsely cellular.

### ≥97% CD-1 immunohistochemistry

For Nissl staining, 40 µm free-floating sections were cut using a freezing stage microtome (Leica). Nissl staining was performed by mounting sections onto slides and proceeding with staining (Paul et al., 2008) with minor modifications. In brief, each solution step was performed 2× for 2 min, an additional 5-min xylene step was introduced following 100% ethanol during the demyelination stage, 0.2% Cresyl Violet was used to stain sections for 20 min, and differentiation solution was omitted. Slides were placed in xylene following destain procedures and coverslipped with DPX (Sigma-Aldrich). Brightfield images were captured using a Nikon Eclipse Ti2-E microscope. Images were white-balance corrected in FIJI and color corrected in Adobe Photoshop. Sections were chosen and matched based on morphology. Original images are available upon request.

### ≥97% CD-1 MRI and analysis

#### Brain preparation

Mice were anesthetized with Beuthanasia-D solution and transcardially perfused with 1× PBS+1000 USP units/ml heparin+2 mM ProHance (Gadoteridol; Bracco Diagnostics), followed by 10% phosphate-buffered formalin+2 mM ProHance. Skulls (with intact brains) were removed and postfixed overnight in 10% phosphate-buffered formalin+2 mM ProHance, followed by storage in 1× PBS+2 mM ProHance+0.02% sodium azide for a minimum of 1 month (De Guzman et al., 2016).

#### Magnetic resonance imaging

A multi-channel 7.0 Tesla MRI scanner (Agilent Inc., Palo Alto, CA, USA) was used to image the brains within their skulls. To increase throughput, 16 custom-built solenoid coils were used to image the brains in parallel (Bock et al., 2005; Lerch et al., 2011).

#### Anatomical scan

In order to detect volumetric changes, the following parameters were used for the MRI scan: T2 weighted, 3D fast spin-echo sequence, with a cylindrical acquisition of k-space, a repetition time (TR) of 350 ms, time to echo (TE) of 12 ms per echo for six echoes, field of view equal to 20×20×25 mm^3^ and matrix size equal to 504×504×630. Our parameters output an image with 0.040 mm isotropic voxels. The total imaging time was ∼14 h (Spencer Noakes et al., 2017).

#### MRI registration and analysis

To visualize and compare any changes in the mouse brains, the images were linearly (six followed by 12 parameters) and non-linearly registered together. Registrations were performed with a combination of mni_autoreg tools (Collins et al., 1994) and advanced normalization tools (ANTS) (Avants et al., 2008, 2011). Once the registration was complete, all scans were then re-sampled with the appropriate transform and averaged to create a population atlas representing the average anatomy of the study sample. The result of the registration is to have all images deformed into alignment with each other in an unbiased fashion. This allows for the analysis of the deformations needed to take each individual mouse's anatomy into this final atlas space, with the goal being to model how the deformation fields relate to genotype (Lerch et al., 2008; Nieman et al., 2006). The Jacobian determinants of the deformation fields are calculated as measures of volume at each voxel, which allows volume differences to be calculated by warping a pre-existing classified MRI atlas onto the population atlas. This atlas includes 182 segmented structures encompassing cortical lobes, large white matter structures (i.e. corpus callosum), ventricles, cerebellum, brain stem and olfactory bulbs (Dorr et al., 2008; Richards et al., 2011; Steadman et al., 2014; Ullmann et al., 2013), which allows regional assessment of the dataset. Further, these measurements can be examined on a voxel-wise basis in order to localize the differences found within regions or across the brain.

### Macroscopic image collection and analysis

Macroscopic images were collected using either an iPhone XS (Fig. 6A,B, Fig. 7A) or Leica dissection microscope with MacroFire camera (Fig. 5, Fig. 6C,D). Images were captured with a scale reference, and color and/or brightness were corrected in Adobe Photoshop. Original images are available upon request.

### Statistical analysis

All statistical analysis was conducted using R (www.cran.r-project.org). Statistical tests used for each experiment are noted where relevant. Mixed effects modeling was performed using the lme4 package (Bates et al., 2015), and statistical testing of the models was performed using the lmerTest package (Kuznetsova et al., 2017). Male and female data were modeled separately. For repeated measures models, random slope and random intercept models were used, with time and genotype treated as fixed effects and mouse identity treated as a random effect. For single measurement models, random intercept models were used, with genotype treated as a fixed effect and litter treated as a random effect, unless otherwise specified. Multiple comparisons in the MRI analysis were controlled for using the FDR (Genovese et al., 2002). *P*-values or FDRs under 0.05 were considered significant.

## Supplementary Material

10.1242/dmm.049752_sup1 Supplementary information
