# Supplementary material for: 17q12 deletion syndrome mouse model shows defects in craniofacial, brain and kidney development, and glucose homeostasis
Source: Dis Model Mech. 2022 Dec 13;15(12):dmm049752. doi: 10.1242/dmm.049752 (PMC10655816; doi:10.1242/dmm.049752)
Supplement: Supplementary information [file dmm-15-049752-s1.pdf]

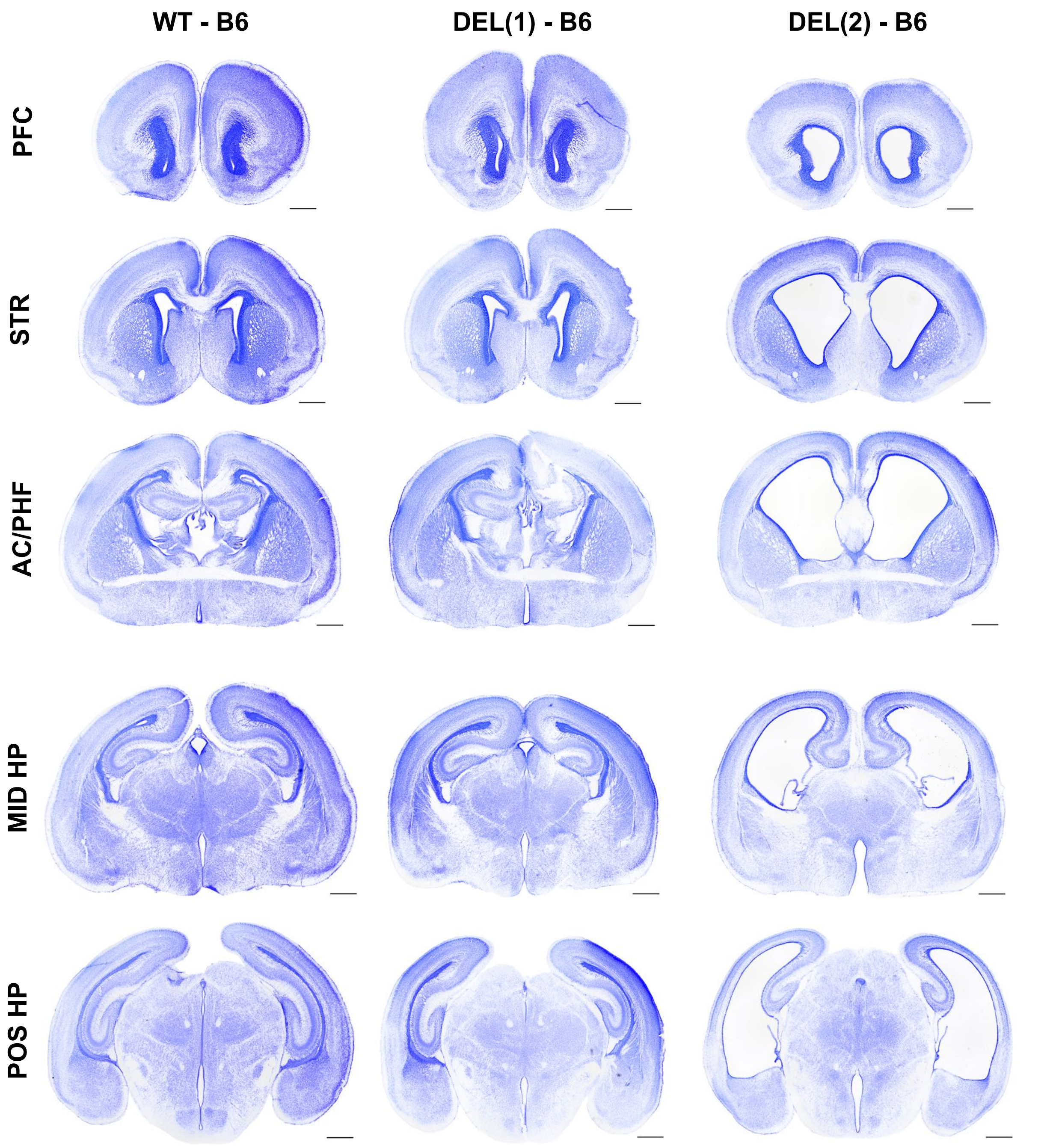

Fig. S1. Neonatal 17q12Del brain malformations include reduced brain width, hippocampal hypoplasia, and ventricular dilatation. Nissl-stained serial sections of B6 P0 brains from wild-type and 17q12Del brains (scored 1 and 2, see Figure 5) reveal profound abnormalities. 17q12Del(1) brains (center) are reduced in width whereas anterior sections are increased in height, but major cortical and subcortical structures are intact. In contrast, 17q12Del(2) brains (right) have enlarged ventricles, thinned cortex, and hippocampal hypoplasia. Sections are labeled to indicate landmark structure in the approximate region of coronal section; PFC – prefrontal cortex, STR – striatum, AC/PHF – anterior commissure/prehippocampal formation, MID HP – mid-hippocampus, POS HP – posterior hippocampus. Scale bar is 0.5mm.

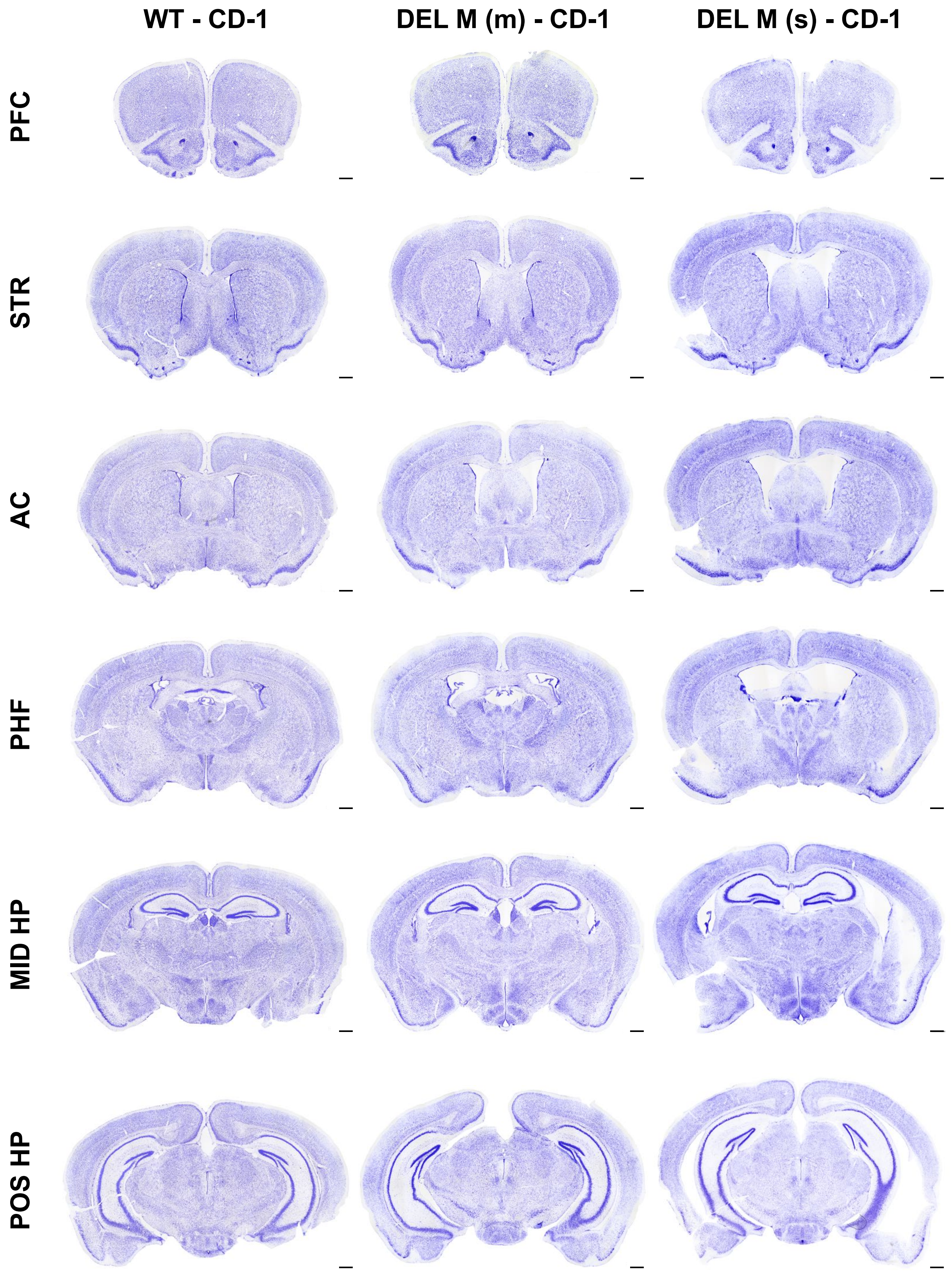

Disease Models & Mechanisms • Supplementary information

Fig. S2. Adult male 17q12Del CD-1 brain malformations vary in severity. Nissl-stained serial sections from representative CD-1 6-week-old brains from wild-type and 17q12Del male mice. (m) brain sections are representative of a mild deletion phenotype, (s) brain sections are representative of a severe deletion phenotype. 17q12Del(m) brains (center) have mild ventricular dilatation, mild callosal thinning, and increased brain height. 17q12Del(s) brains (right) have profound ventricular dilatation, severe callosal thinning, increased brain height, and severe posterior cortical thinning. Sections are labeled to indicate landmark structure in the approximate region of coronal section; PFC – prefrontal cortex, STR – striatum, AC – anterior commissure, PHF – prehippocampal formation, MID HP – mid-hippocampus, POS HP – posterior hippocampus. Scale bar is 0.5mm.

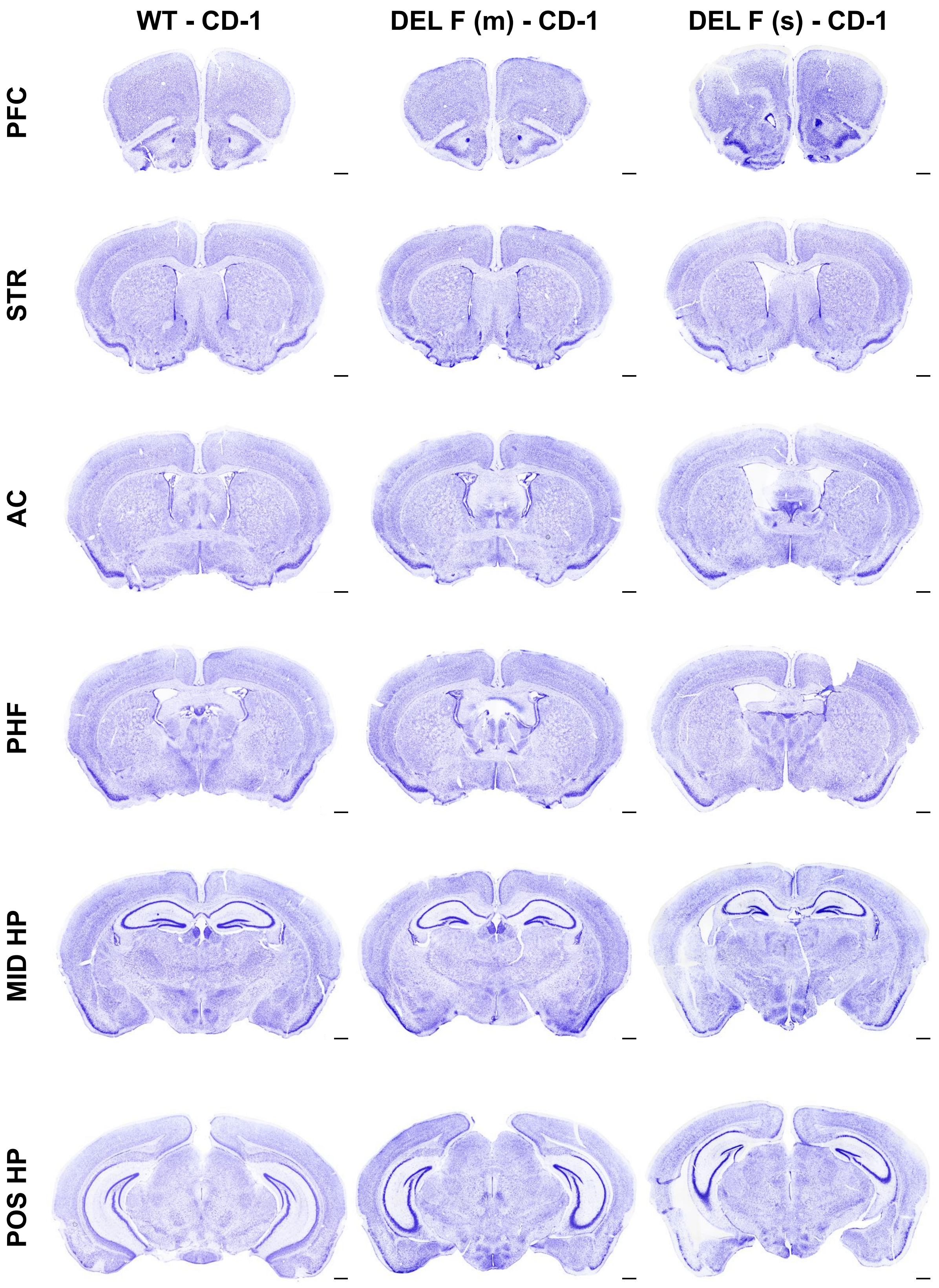

Disease Models & Mechanisms • Supplementary information

Fig. S3. Adult female 17q12Del CD-1 brain malformations are less prominent than in males. Nissl-stained serial sections from representative CD-1 6-week-old brains from wild-type and 17q12Del female mice. (m) brain sections are representative of a mild deletion phenotype, (s) brain sections are representative of a severe deletion phenotype. The 17q12Del(m) brain (center) has few macroscopic anomalies, though the brain is smaller than wild-type. The 17q12Del(s) brain (right) has asymmetric ventricular dilatation, moderate callosal thinning, increased brain height, and severe posterior cortical thinning. Sections are labeled to indicate landmark structure in the approximate region of coronal section; PFC – prefrontal cortex, STR – striatum, AC – anterior commissure, PHF – prehippocampal formation, MID HP – mid-hippocampus, POS HP – posterior hippocampus. Scale bar is 0.5mm.

Disease Models & Mechanisms • Supplementary information

Table S1. Animal numbers for each experiment. Tabulation of all animals used for experiments in this study. Litters for each genotype indicates number of litters where each sex-genotype combination was found.

Figure	Sex	Genotype	Litters	Background	Age	N
2B*	M+F	WT	8	100% B6	≥P21	41
	M	DEL	0	100% B6	≥P21	0
	F	DEL	0	100% B6	≥P21	0
	M+F	WT	13	75% B6:25% CD-1	≥P21	52
	M	DEL	2	75% B6:25% CD-1	≥P21	2
	F	DEL	2	75% B6:25% CD-1	≥P21	2
	M+F	WT	6	50% B6:50% CD-1	≥P21	62
	M	DEL	2	50% B6:50% CD-1	≥P21	2
	F	DEL	1	50% B6:50% CD-1	≥P21	1
	M+F	WT	10	25% B6:75% CD-1	≥P21	70
	M	DEL	7	25% B6:75% CD-1	≥P21	16
	F	DEL	9	25% B6:75% CD-1	≥P21	17
	M+F	WT	39	≥97% CD-1	≥P21	260
	M	DEL	35	≥97% CD-1	≥P21	69
	F	DEL	30	≥97% CD-1	≥P21	61
	M+F	WT	8	≥75% B6	P0	47
	M	DEL	5	≥75% B6	P0	13
	F	DEL	5	≥75% B6	P0	11
	M+F	WT	22	≥75% B6	E7-14	140
	M	DEL	20	≥75% B6	E7-14	56
	F	DEL	21	≥75% B6	E7-14	55
3A	M	WT	5	≥97% CD-1	P0-P42	12
	M	DEL	4	≥97% CD-1	P0-P42	11
3B	F	WT	4	≥97% CD-1	P0-P42	12
	F	DEL	4	≥97% CD-1	P0-P42	10
3C-F, 4	M	WT	6	≥97% CD-1	P42-P56	13
	M	DEL	8	≥97% CD-1	P42-P56	15
	F	WT	4	≥97% CD-1	P42-P56	13
	F	DEL	5	≥97% CD-1	P42-P56	13
5	M	WT	3	≥75% B6	E8.5	8
	M	DEL	3	≥75% B6	E8.5	5
	F	WT	3	≥75% B6	E8.5	8
	F	DEL	3	≥75% B6	E8.5	8
	M	WT	3	≥75% B6	E9.5	9
	M	DEL	3	≥75% B6	E9.5	7
	F	WT	3	≥75% B6	E9.5	6

Disease Models & Mechanisms • Supplementary information

	F	DEL	3	≥75% B6	E9.5	6
	M	WT	5	≥75% B6	E10.5	10
	M	DEL	5	≥75% B6	E10.5	14
	F	WT	5	≥75% B6	E10.5	15
	F	DEL	5	≥75% B6	E10.5	11
	M	WT	2	≥75% B6	E12.5	3
	M	DEL	2	≥75% B6	E12.5	5
	F	WT	2	≥75% B6	E12.5	4
	F	DEL	2	≥75% B6	E12.5	4
6, S1	M	WT	7	≥75% B6	P0	26
	M	DEL	5	≥75% B6	P0	13
	F	WT	8	≥75% B6	P0	21
	F	DEL	5	≥75% B6	P0	11
7, S2, S3[†]	M	WT	8	≥97% CD-1	P42-P56	18
	M	DEL	7	≥97% CD-1	P42-P56	21
	F	WT	6	≥97% CD-1	P42-P56	17
	F	DEL	6	≥97% CD-1	P42-P56	19
8[‡]	M	DEL	7	≥97% CD-1	P42-P56	16
	M	DEL	7	≥97% CD-1	P42-P56	15

* All animals used in this study are included in Figure 2B

[†] Animals used for Figures 7, S2, and S3 were also used for Figures 3C-3F and 4.

[‡] Animals used for Figure 8 were also used for Figures 3C-3F, 4, 7, and S2

Disease Models & Mechanisms • Supplementary information

Table S2. Severity score categories. ≥75% C57BL/6N or CD-1 animals were collected at indicated stage and assessed for abnormal phenotypes. Abnormalities were binned into the above categories and each pup was assigned a severity score. For embryonic animals, Theiler staging was performed with reference to major developmental hallmarks. Developmental delay was determined in reference to average wildtype TS per litter. In brief, for stages where some deletion embryos were developmentally delayed, TS12a and TS12b were differentiated by allantois contact with the embryo, TS13 and TS14 were differentiated by appearance of the forelimb bud and position of the brachial arches, and TS16 and TS17 were differentiated by the shape of the otic vesicle. See Figure 3 for representative embryos for each category. For P0 animals, see Figure 4 for representative offspring for each category, and Figure S1 for representative Nissl sections from category 1 and 2 offspring. For CD-1 animals, see Figure 5 for craniofacial features, and Figures S2 and S4 for representative Nissl sections from male and female deletion animals.

Strain	Stage	Score	Phenotypes and Criteria
B6	E8.5	(none)	TS12b-TS14, stage-appropriate features, no abnormalities noted
B6	E8.5	Mild (m)	TS12b-TS13, with two or fewer of the following abnormalities:<br>-absence of optic envagination<br>-deficient forebrain development<br>-increased relative rhombencephalon size<br>-absence of clear tectum mesencephali
B6	E8.5	Severe (s)	TS12a (i.e., developmental delay), with any of the following abnormalities, OR TS12b, with more than two of the folowing:<br>-absence of optic invagination<br>-deficient forebrain development<br>-increased relative rhombencephalon size<br>-absence of clear tectum mesencephali
B6	E9.5	(none)	TS15-TS16, stage-appropriate features, no abnormalities noted
B6	E9.5	Mild (m)	TS15, with two or fewer of the following abnormalities:<br>-absence or deficiency of optic vesicle<br>-abnormality in mesencephalic structure or size<br>-abnormality of prosencephalic structure or size
B6	E9.5	Severe (s)	TS14 (i.e., developmental delay), with any of the following abnormalities:<br>-absence or deficiency of optic vesicle/invagination<br>-abnormality in mesencephalic structure or size<br>-abnormality of prosencephalic structure or size<br>OR:<br>TS15, with two or more of the following abnormalities:<br>-absence or deficiency of optic vesicle<br>-abnormality in mesencephalic structure or size<br>-abnormality of prosencephalic structure or size<br>-overtly deficient head size/development relative to embryo stage
B6	E10.5	(none)	TS17-TS18, stage appropriate features, no abnormalities noted
B6	E10.5	Mild (m)	TS17-TS18, with at least one of the following abnormalities:<br>-deficient and/or underdeveloped but present telencephalic vesicles<br>-underdevelopment of the lens vesicle<br>-underdevelopment of the metencephalon but presence of the isthmus<br>-underdevelopment of the olfactory pit
B6	E10.5	Severe (s)	TS16-TS17, with overt abnormalities of the head and/or brain, including two or more of the following:<br>-absence or severe malformation of the telencephalon and/or diencephalon<br>-severe malformation and/or under-differentiation of the midbrain or hindbrain<br>-absence of the lens vesicle or optic invagination

Disease Models & Mechanisms • Supplementary information

B6	E12.5	(none)	TS20-TS21, stage appropriate features, no abnormalities noted
B6	E12.5	Mild (m)	TS20-TS21, with at least one of the following abnormalities:<br>-underdevelopment or absence of the lens<br>-underdeveloped but identifiable brain and/or head structure (i.e. observable telencephalic vesicles)
B6	E12.5	Severe (s)	TS20-TS21, with overt abnormalities of the head and/or brain, including two or more of the following:<br>-absence or severe malformation of the telencephalon and/or diencephalon<br>-severe malformation and/or under-differentiation of the midbrain or hindbrain<br>-absence of the lens vesicle or optic invagination
B6	P0	0	No abnormalities noted
B6	P0	1	Any combination of the following: shortened snout/nasal bones, reduced brain size, “hollow” brain, mild eye abnormalities
B6	P0	2	Features of score 1, plus: overt asymmetry of nose and/or maxilla, profound underdevelopment of one eye
B6	P0	3	Any combination of the following: incomplete closure of the maxilla, incomplete closure of the skull, unilateral malformations of the cortical hemispheres
B6	P0	4	Features of score 3, plus any combination of: profound brain abnormalities (lack of differentiation, incomplete closure of the neural tube, protrusions through the skull), lack of all forebrain structures
CD-1	6-8wk	WT (none)	No abnormalities noted
CD-1	6-8wk	Mild (m)	No craniofacial abnormalities noted
CD-1	6-8wk	Severe (s)	Presence of nasal asymmetry or depression

Table S3. MRI absolute volume results and statistics.

Click here to download Table S3

Table S4. MRI relative volume results and statistics.

Click here to download Table S4

Table S5. MRI regions and developmental structural ontogeny classifications.

Click here to download Table S5

Table S6. MRI structural ontogeny region volume results and statistics.

Click here to download Table S6

Disease Models & Mechanisms • Supplementary information
